# Non-reciprocal response in silicon photonic resonators integrated with 2D CuCrP_2_S_6_ at short-wave infrared

**DOI:** 10.1038/s41377-025-01826-w

**Published:** 2025-04-09

**Authors:** Ghada Dushaq, Solomon Serunjogi, Srinivasa R. Tamalampudi, Mahmoud Rasras

**Affiliations:** 1 Department of Electrical and Computer Engineering, New York University Abu Dhabi, P.O. Box 129188, Abu Dhabi, UAE; 2 NYU Tandon School of Engineering, New York University, Brooklyn, NY USA

**Keywords:** Silicon photonics, Optoelectronic devices and components

## Abstract

Achieving non-reciprocal optical behavior in integrated photonics with high efficiency has long been a challenge. Here, we demonstrate a non-reciprocal magneto-optic response by integrating multilayer 2D CuCrP_2_S_6_ (CCPS) onto silicon microring resonators (MRRs). Under an applied magnetic field, the CCPS intralayer ferromagnetic ordering, characterized by easy-plane magneto-crystalline anisotropy, induces asymmetrical modal responses in the clockwise (CW) and counterclockwise (CCW) light propagation directions. The proposed configuration achieves a low insertion loss ranging from 0.15 dB to 1.8 dB and a high isolation ratio of 28 dB at 1550 nm. Notably, it exhibits a significant resonance wavelength splitting of 0.4 nm between the counter propagation directions, supporting a 50 GHz optical bandwidth. Operating directly in the transverse electric (TE) mode, it aligns with the main polarization used in silicon photonics circuits, eliminating the need for additional polarization management. The device is ultra-compact, with a 2D flake interaction length ranging from 22 µm to 55 µm and a thickness between 39 nm and 62 nm. Its operation range covers the entire C-band with a bandwidth of up to 100 nm. These attributes make our hybrid CCPS/Si device ideal for advanced non-reciprocal optical applications in the short-wave infrared (SWIR) spectrum, crucial for enhancing the resilience of optical systems against back-reflections.

## Abstract (web summary)

A hybrid CCPS/Si microring enables non-reciprocal TE-mode operation with 28 dB isolation, 50 GHz bandwidth, and 0.4 nm resonance splitting in the C-band.

## Graphical abstract

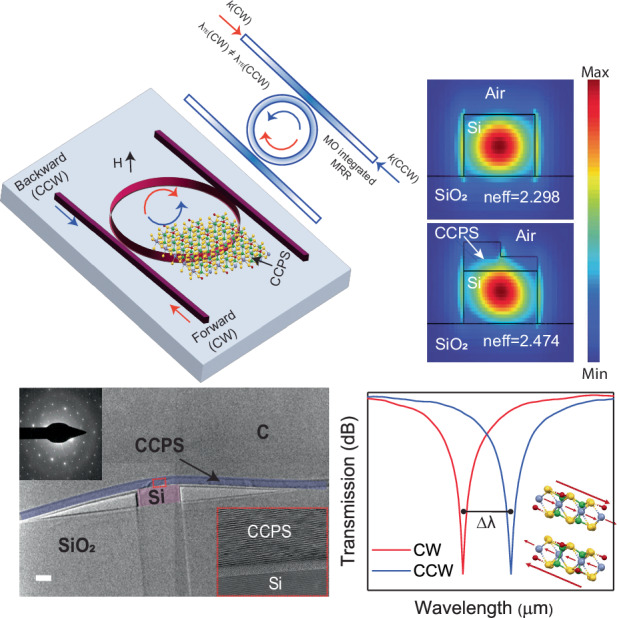

## Introduction

Non-reciprocal photonic devices, which break the Lorentz reciprocity of light propagation, are essential for the development of advanced integrated optical devices^1–4^. These devices enable critical functionalities such as signal isolation and directional transmission, which are pivotal in applications ranging from telecommunications to quantum computing^5,6^. While electrical non-reciprocity has been effectively achieved through the use of semiconductor p–n junctions, optical non-reciprocity (ONR) presents significant challenges^7^. Achieving ONR pivots on combining innovative device design as well as the development and integration of tailored materials.

Several approaches have been explored to break Lorentz reciprocity, including nonlinear optical effects^5,8^ and spatio-temporal modulation^9,10^. Among these, techniques, utilizing magneto-optical (MO) effects are particularly compelling^3,4,11–14^. Conventionally, MO devices exploit mode conversion through the Faraday effect, a common approach in bulk nonreciprocal devices^15,16^. However, on-chip waveguides, characterized by their inherent birefringence, generally favor designs that employ nonreciprocal phase shifts (NRPS)^17^. Such configurations include ring resonators, multimode interferometers, and Mach–Zehnder interferometers (MZIs)^3,4,18–22^. For waveguide integration in the short-wavelength infrared (SWIR) region, yttrium iron garnets (YIG) doped with Bi or Ce to boost Faraday rotation are particularly effective^11,23^. However, the integration of these magneto-optical garnets with semiconductor substrates faces challenges, such as substantial lattice mismatches and thermal incompatibilities^4,24,25^. These obstacles hinder achieving monolithic integration with strong Faraday rotation and minimal transmission loss^26^. Moreover, nonreciprocal devices utilizing these materials typically have large footprints, ranging from millimeters to centimeters, posing significant barriers to large-scale and cost-effective integration^11^. Thus, there is a pressing need for on-chip integrated nonreciprocal photonic devices that combine a suitable material with a compact footprint and robust performance.

In contrast to traditional 3D magnetic thin films, two-dimensional (2D) materials are emerging as alternatives for fabricating on-chip tunable optical components, such as electro-optic modulators, photodetectors, and filters^27–31^. Their pronounced quantum confinement effects and high refractive indices facilitate strong light–matter interactions, markedly influencing their optical properties in response to external stimuli^32,33^. Importantly, the layered architecture of 2D materials, characterized by covalent in-plane bonds and van der Waals forces between planes, enables straightforward integration onto diverse substrates, circumventing lattice matching issues and simplifying the fabrication process^28,29^. A recent study on a hybrid graphene/silicon magneto-optical isolator has been developed for integration within the SWIR region^26^. This device capitalizes on spin–orbit interactions coupled with graphene’s unique properties to achieve an extinction ratio of 45 dB and an insertion loss of 12 dB at 1552 nm wavelength. However, the practical deployment of such devices is constrained by the need for cryogenic temperatures and strong magnetic fields to overcome graphene’s relatively weak magneto-optical effect. Furthermore, an optical isolator has been realized in graphene oxide utilizing optical symmetry-breaking mechanisms, including photo-thermal effects. This hybrid system facilitates transverse electric mode NRPS without the need for structural asymmetry^34^.

Recent advances have heightened interest in 2D multiferroic materials^35,36^, notably CuCrP_2_S_6_ (CCPS), recognized for their optical transparency in the SWIR spectrum, multiple ferroic orders, and a refractive index (3.2 RIU at 1550 nm) with relatively small contrast to silicon (3.47 RIU at 1550 nm)^37,38^. CCPS, a type-II multiferroic, intriguingly couples spin, valley, and electric dipoles. This coupling emerges from the unique interplay between its two distinct sublattices: the ferromagnetic order arises predominantly from chromium (Cr) atoms, while copper (Cu) atoms contribute to the ferroelectric polarization^39–42^. Additionally, previous studies have offered experimental and theoretical support for the presence of magnetoelectric coupling in CCPS, emphasizing the pivotal role of spin–orbit coupling in connecting electric dipoles and spins^37,40,41,43,44^. This interplay suggests that magnetic fields can induce electric polarization and that electric fields can modulate magnetic ordering, even at the monolayer level. While much research has focused on CCPS’s memristive properties for data storage and neuromorphic computing applications^42,45,46^, its potential in integrated photonics for efficient and chip-scale non-reciprocal devices remains underexplored. Integrating CCPS could significantly advance the development of photonic circuits by leveraging its magneto-optical properties to manage light directionally.

In this study, we explore the magneto-optic response of multi-layer CCPS integrated onto SiPh microring resonators (MRR) at the SWIR regime. Leveraging the intralayer ferromagnetic ordering within CCPS, characterized by easy-plane magnetocrystalline anisotropy, we clarify the physics underpinning significant enhancements in magneto-optical properties and non-reciprocal optical phenomena. This magnetic anisotropy enables modal asymmetry under a magnetic field, optimizing the device for non-reciprocal behavior with minimal optical losses. In particular, CCPS exhibits magneto-optic properties when subjected to an external magnetic field, which induces a change in the phase of light depending on its direction of propagation (i.e., clockwise or counterclockwise). This phase shift occurs because the magnetic field alters the polarization-dependent refractive index of the CCPS layer, which interacts with the evanescent field of the light confined within the waveguide. The interaction between the CCPS and the guided optical mode gives rise to the observed nonreciprocal behavior. The CCPS-loaded MRR shows strong light-matter interaction and depicts a low insertion ranging from 0.15 to 1.8 dB with an isolation ratio of 28 dB. The results are obtained at 1550 nm wavelength using a compact interaction length of just 22–55 µm and a 2D flake thickness ranging from 39 to 62 nm. The device supports a 50 GHz optical bandwidth (resonance splitting of 0.4 nm) and maintains low dispersion across a broad operational wavelength range of up to 100 nm, surpassing traditional Faraday-effect-based devices.

Furthermore, its operation in the TE-polarization mode is aligned with the primary polarization used in silicon photonics. This performance eliminates the need for additional components like polarization rotators, thus simplifying integration and enhancing efficiency. Therefore, these features position the CCPS/Si integration as a robust solution for SWIR nonreciprocal devices and optical isolators, crucial components for mitigating back-reflection in advanced optical systems.

## **Results**

### Non-reciprocal photonics: overview and principles

**Fig. 1:**
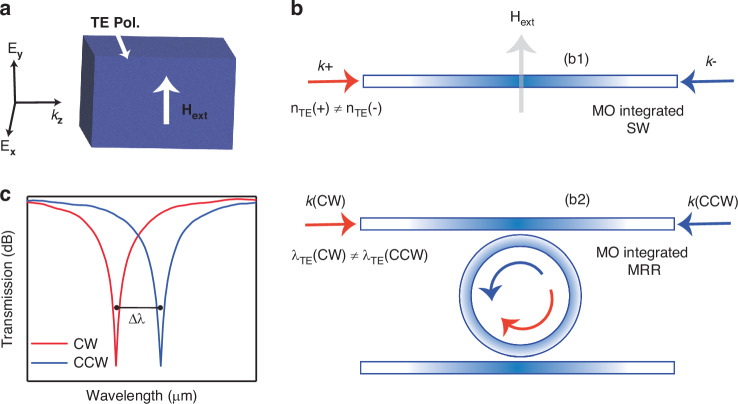
Non-reciprocal phase shift (NRPS) in photonic structure. **a** Voigt configuration, illustrating the wave number (*k*_*z*_) of light propagating in the *z*-direction within a magneto-optic medium. **b** Schematic of a nonreciprocal magneto-optical waveguide (top panel, **b1**) depicting non-degenerate effective indices for forward and backward propagating TE-polarized light. The bottom panel (**b2**) illustrates a nonreciprocal magneto-optical resonator, showing non-degenerate resonant frequencies between clockwise and counterclockwise TE optical modes. In the waveguide, *k*+ and *k*− represent the wave numbers of the propagating mode in the two counter-propagation directions. **c** Transmission spectra of the integrated magneto-optic material in a ring resonator, demonstrating the resonance wavelength split (Δ*λ*) under a static magnetic field

In magneto-optical integrated devices, nonreciprocal phase shifts (NRPS) primarily result from field asymmetry in materials configured under Voigt geometry^17,47,48^. Figure 1a illustrates Voigt geometry where the magnetization of the magneto-optic medium is perpendicular to the direction of light propagation. This unique alignment influences the light’s behavior as it traverses the medium in different directions, primarily due to the magnetic field’s impact on the material’s optical properties highlighted by the material’s anisotropy and mathematically expressed using the permittivity tensor^49,50^.

NRPS varies with the light’s direction and polarization; for instance, the behavior of the transverse magnetic (TM) and transverse electric (TE) modes depends on whether the magnetic field aligns in-plane or out-of-plane with respect to the waveguide. This leads to different propagation constants for the forward (*β*+) and backward (*β*−) traveling light^51^. The discrepancy between these constants (Δ*β* = *β*+−*β*−) changes according to the direction of magnetization relative to the light propagation. This variability introduces a fundamental aspect of reconfigurability in our study. The influence of the waveguide’s structural design on the NRPS modifies the propagation constants for TM and TE modes, and this dynamic is mathematically detailed as follows^11,52^:

1 $$\Delta \beta \left({\rm{TM}}\right)=\frac{2{\beta }^{{{\rm {TM}}}}}{\omega {\varepsilon }_{0}N}\,\iint \frac{\gamma }{{n}_{0}^{4}}{H}_{x}{\partial }_{y}{H}_{x}{{ {d}}x{ {d}}y}$$

2 $$\Delta \beta \left({\rm{TE}}\right)=\frac{2\omega {\varepsilon }_{0}}{{\beta }^{{{\rm {TE}}}}N}\,\iint \gamma {E}_{x}{\partial }_{x}{E}_{x}{{ {d}}x{ {d}}y}$$

Here, the propagation constants for the fundamental transverse magnetic (TM) and transverse electric (TE) modes are denoted by ${\beta }^{{{\rm {TM}}}}$ and ${\beta }^{{{\rm {TE}}}}$, respectively. The angular frequency of the wave is represented by *ω*, while *ε*_0_ signifies the vacuum dielectric constant. The term *N* refers to the power flux along the direction of light propagation, labeled as the *z*-direction, here the power flux refers to the integral of the Poynting vector over a surface. The parameters *n*_0_ and *γ* denote the refractive index and the off-diagonal component of the permittivity tensor of the magneto-optic (MO) materials, respectively. Additionally, *H*_*x*_ and *E*_*x*_ correspond to the magnetic and electric field components of the fundamental TM and TE modes, respectively. The partial derivatives *d*_*x*_ and *d*_*y*_ of the electric field components *E*_*x*_ and the magnetic field components *H*_*x*_ account for the spatial variation with respect to the coordinates *x* and *y*. These parameters collectively describe the electromagnetic behavior within the MO materials under investigation.

Utilizing the Voigt configuration, we investigate the integration of a magneto-optic CCPS material within a microresonator. The CCPS is positioned to partially cover an arc of the resonator waveguide, making it magneto-optically active and breaking its *X*-axis symmetry, as further detailed in Supplementary Note 1. The operational principle of this nonreciprocal optical resonator involves using the magneto-optical effect to eliminate the frequency degeneracy typically observed between the forward and backward-propagating light in optical resonators. In standard optical resonators without magneto-optical properties, the resonant frequencies for both propagation directions remain the same^53^. Previous reports have demonstrated that the degeneracy of resonances can be lifted due to coupling between forward- and backward-propagating modes, a phenomenon known as Autler–Townes splitting, which arises from backscattering^54^. However, by incorporating the nonreciprocal phase shift (NRPS) effect, the resonance wavelengths become directionally dependent.

For further clarity, refer to Fig. 1b. In a straight optical waveguide made of magneto-optical materials, the TE polarized modes undergo an NRPS when an external magnetic field is applied perpendicular to the light propagation direction. This shift leads to distinct effective indices (${n}_{\text{eff}})$ for modes traveling forward and backward. As illustrated in Fig. 1b, when this effect is applied within a ring resonator structure it results in distinct resonant wavelengths for the clockwise (CW) and counter-clockwise (CCW) directions. This effect is depicted in Fig. 1c highlighting the resonance wavelength split (RWS) between CW and CCW light propagation. The RWS not only reveals the differences in effective indices ($\varDelta {n}_{\text{eff}}$) but also shows a divergence in their resonance wavelengths. The magnitude of the RWS (Δ*λ*) is directly related to the alterations in the light propagation constant ($\Delta \beta$) and the effective refractive index, as follows^4,13,18^:

3 $$\Delta \beta =\frac{2\pi \Delta \lambda }{{\rm{FSR}}{L}_{\text{MO}}}$$

4 $$\Delta \lambda =\lambda (\varDelta {n}_{\text{eff}})/{n}_{\text{g}}$$

where *L*_MO_ refers to the interaction length of the MO waveguide, FSR is the free spectral range of the micro ring resonator and *n*_g_ is the group index.

### Device design and fabrication

**Fig. 2:**
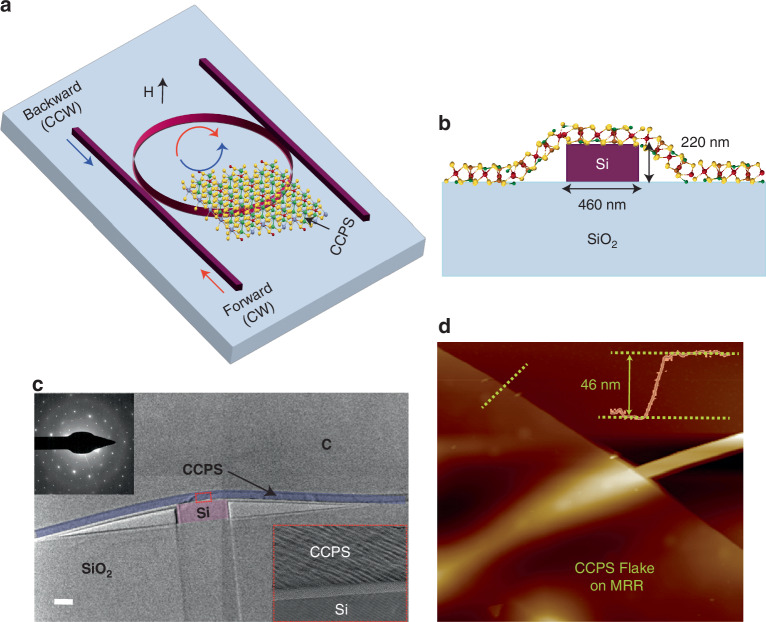
Device design and CCPS/SiPh integration. **a** 3D schematic of the silicon microring resonator (MRR) design with integrated CCPS. **b** Cross-section illustrating the design and fabrication parameters. **c** False colored transmission electron microscopy (TEM) cross-section image of the hybrid integration with a selected area diffraction pattern (SADP) of CCPS; the red dashed square indicates the interface between the silicon waveguide and CCPS, C refers to the protective carbon layer. **d** Atomic force microscopy (AFM) image scan, with the yellow dashed line showing a CCPS thickness of ~46 nm. Scale bars for (**c**) and (**d**) are 0.2 µm, 10 nm^-1^ and 0.5 µm, respectively

Figure 2a, b illustrates the architecture for the hybrid integration of multilayer CuCrP_2_S_6_ (CCPS) with silicon photonics (SiPh) circuits. The structure utilizes silicon-on-insulator (SOI) wafers, comprising a 220 nm top silicon device layer over a 2 µm buried oxide layer. The add-drop microring resonator (MRR) features a 460 nm-wide waveguide with a 45 µm ring radius. A 100 nm gap between the bus waveguide and the resonator allows for the coupling of light, with critical coupling achieved at the through port output, optimizing the extinction ratio. In this configuration, an application of an external magnetic field perpendicular to the plane of the ring resonator induces a non-reciprocal phase shift.

The integration of CCPS onto the ring waveguide enables precise control of light propagation through the “add” and “through” ports by modulating the CCPS’s refractive index via magneto-optic tuning. Light in the waveguide is evanescently coupled to the CCPS layer, and the coupling strength is modulated by the CCPS thickness.

The multilayer CCPS flakes were obtained through mechanical exfoliation from commercially available bulk crystals sourced from HQ Graphene manufacture. Comprehensive structural characterization, including atomic force microscopy (AFM), energy dispersive X-ray spectroscopy (EDX), X-ray photoelectron spectroscopy (XPS), and transmission electron microscopy (TEM) with diffraction patterns, as well as optical properties such as refractive index (n), extinction coefficient ($\kappa$), bandgap, and material absorption, have been detailed in our previous work^38^. Additional information is provided in the supporting information (Figs. S1–S4). For device integration, CCPS flakes with varying thicknesses ranging from ~39 to ~127 nm were systematically transferred onto the microring resonator (MRR) structure using a deterministic dry transfer process^28,29,55,56^. Details of the fabrication process and deterministic transfer process can be found in the method section and Supplementary Note 4 on material integration and device testing.

Figure 2c presents the morphology of the hybrid Si/CCPS MRR via a transmission electron microscopy (TEM) cross-section image. The CCPS flakes align well with the underlying photonic structure, promoting effective light/CCPS coupling. The inset in Fig. 2c reveals a thin layer of SiO_2_ at the interface between the Si waveguide and CCPS, attributed to the oxidation of Si prior to the CCPS transfer.

To quantitatively determine the thickness of the transferred flakes, atomic force microscopy (AFM) was employed, with results shown in Fig. 2d. Additional insights into AFM scans, cross-section images, and 3D reconstructed images highlighting strain effects in our devices can be found in our earlier work^38^.

### Device characterization

#### Passive optical testing

**Fig. 3:**
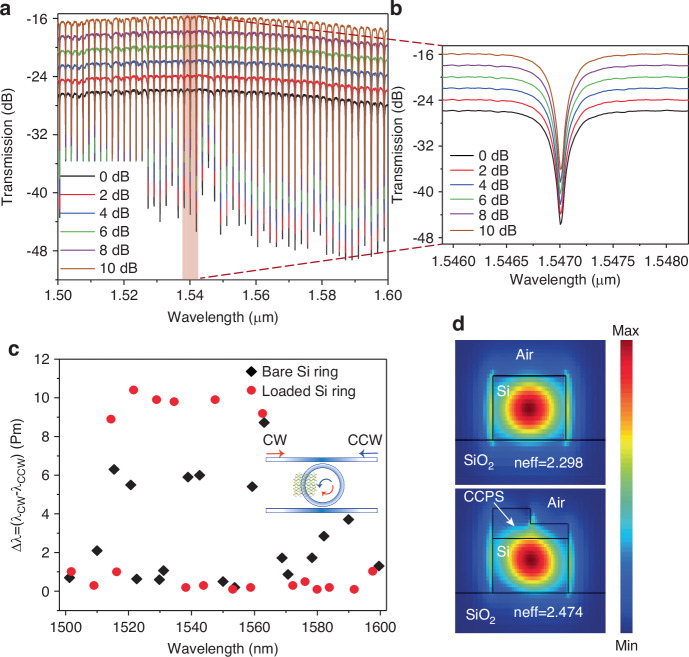
Passive optical properties of hybrid Si/CCPS MRR. **a** Transmission spectra spanning 1500–1600 nm of the MRR at varying input optical powers. **b** Detailed view of the resonance peak, illustrating minimal thermal dissipation from light propagation within the CCPS-integrated MRR. **c** Variations in the position of the resonance peak for forward (CW) and backward (CCW) light propagation in the absence of a magnetic field. **d** Electric field intensity profiles for TE modes at 1550 nm are shown for a bare silicon waveguide (top panel) and a silicon waveguide covered with a non-uniform CCPS layer of ~67 nm average thickness (bottom panel)

To experimentally verify the non-reciprocal phase shift in hybrid integrated devices, we initially examined the passive optical transmission properties of the CCPS-loaded MRR without any magnetic field influence. These baseline measurements underpin the operational performance of the device, which we elaborate on in the subsequent section. A tunable continuous-wave laser emitting at 1550 nm shortwave infrared (SWIR) wavelengths is used to evaluate the performance of the resonator. A TE-polarized light was coupled into the MRR via a lensed fiber and the transmitted light was then collected by another lensed fiber and measured with a power meter. To control and evaluate the impact of the laser input power on the Si/CCPS chip, we systematically adjusted the input power from 0.66 mW (−1.8 dBm) to 10 mW (10 dBm). Figure 3a, b illustrates the transmitted resonance peak positions. As shown in these figures, the transmission spectra remain unchanged across different power levels (refer to the supporting information Fig. S5 for the transmission spectra of the bare silicon ring resonator under varying power conditions). This constancy suggests negligible thermal dissipation effects from the light propagation within the CCPS-integrated MRR. Consequently, we limited the laser power in all further measurements to a maximum of 10 mW (10 dBm). It is important to note that the 10 dBm refers to the laser input power and not the power received by the CCPS/Si-MRR, which is −2 dBm adjusted by 12 dB insertion loss per facet.

Furthermore, to establish a baseline for measurement consistency, we quantified the difference in resonance peak positions between forward (CW) and backward (CCW) light propagation in the hybrid Si/CCPS ring resonator before introducing any magnetic fields. As depicted in Fig. 3c, the variation in the resonance wavelength (Δ*λ*) between CW and CCW light propagation across a spectral range from 1500 to 1600 nm was assessed for both the hybrid and the bare silicon resonators. This is achieved by alternating the coupling of laser light into the chip between the input and output ports. The observed differences in both loaded and unloaded rings reached a maximum of approximately 12 ± 1 pm. Ideally, ring resonators are symmetric structures, resulting in identical transmission spectra for both clockwise (CW) and counterclockwise (CCW) propagation. However, these variations are primarily attributed to fabrication tolerances of the chip, minor temperature fluctuations, and the accuracy of peak position determination in the spectral analysis. These figures present data collected from a minimum of five devices, each featuring CCPS layers approximately 45 and 60 nm thick, with an interaction length of ~33 µm. Results from a bare silicon ring are also included for comparison.

Figure 3d illustrates the electric field intensity profiles of the fundamental guided modes for both bare and loaded Si waveguide structures integrated with a non-uniform CCPS layer of approximately 67 nm average thickness calculated at 1550 nm wavelength. The silicon waveguide predominantly supports a quasi-TE mode. Notably, the evanescent field of the mode effectively interacts with the multilayer CCPS flake, with ~5.2% of the optical mode power localized within the CCPS layer. Additional details on the measured optical parameters, the asymmetric refractive index profile arising from the non-uniform coverage and strain in the CCPS layer, and the calculations of the effective refractive index for the two counter-propagating directions are provided in Supplementary Note 1, Figs. S6–S8.

Additional losses, induced by integrating CCPS flakes, were quantified by comparing a reference silicon ring to the hybrid CCPS/Si structure. These losses primarily stem from reflections and scattering at the interface between the passive (air/Si) and hybrid regions, intensified by mode mismatches and flake irregularities. The latter can be reduced by proper tapering between the air and the flake regions. Importantly, absorption in the shortwave infrared (SWIR) spectrum was minimal^38,39,57^. Notably, the optical losses linked to thin flakes of ~39–44 nm on the resonator were minimal, with no significant alteration in the full width at half maximum (FWHM). This balance of low optical losses and effective magneto-optic tuning surpasses the performance of other magneto-optic materials on silicon photonics platforms, typically optimized for transparency in the O- and C-optical bands for various near-infrared magneto-optic applications^4,18,24,58^. Detailed descriptions of these measurements are provided in Fig. S9—Supplementary Note 1 (optical loss analysis).

#### Magneto-optic characteristic

To investigate the magneto-optic response of CCPS, we employed a hybrid silicon-CCPS microring resonator platform. A segment of CCPS measuring 22–55 μm in arc length and 39–62 nm in thickness was integrated into the resonator. This resonator is critically coupled to a silicon bus waveguide, rendering the transmission spectrum of the ring extremely sensitive to minor phase changes within the resonator. We applied a direct current (DC) magnetic field perpendicular to the light propagation direction across the patterned resonator using an electromagnet; detailed information on the electromagnetic setup can be found in Supplementary Note 2 (Fig. S10). It is noteworthy that reversing the external magnetic field’s direction effectively mirrors the light’s propagation direction^18^. Additionally, we examined the dependency of light directionality in a bare silicon resonator under a magnetic field. As shown in Fig. S11, no resonance wavelength splitting (RWS) was observed in silicon, attributed to its diamagnetic nature.

**Fig. 4:**
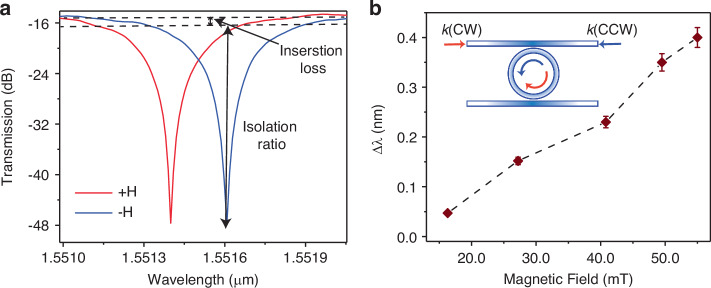
Active detuning under a direct current (DC) magnetic field (Nonreciprocal Measurement). **a** Transmission spectra for the TE mode, recorded with a fixed light propagation direction while reversing the direction of the magnetic field, demonstrating a non-reciprocal resonance shift. **b** Resonance wavelength splitting (RWS), defined as Δ*λ* = *λ*_CW_−*λ*_CCW_, plotted against the applied magnetic field strength for a device with a thickness of ~45 nm and interaction length of 38 µm

Conversely, in the CCPS-loaded ring, the effective refractive index of the optical mode is tuned by the magnetic characteristics of CCPS. Figure 4a presents the transmission spectra for the TE mode in a hybrid CCPS ring resonator, subjected to a magnetic field of 40 mT while being maintained at room temperature with an accuracy of ±0.2 °C. This assessment was validated by repeating each measurement ten times using two methodologies: alternating the direction of light propagation and switching the direct current through the electromagnet. Notably, the transmission spectra from forward (CW-red) and backward (CCW-blue) propagation displayed alternating resonance dips, which were detuned by approximately 0.2 nm, indicating a nonreciprocal phase shift between these modes. The intralayer ferromagnetic ordering in CCPS, characterized by an easy-plane magnetocrystalline anisotropy within the van der Waals basal plane, significantly alters the magnetooptical properties. The magnetic anisotropy in CCPS is a prime factor for exhibiting non-reciprocal optical phenomena due to modal asymmetry when exposed to a magnetic field^40^. Additionally, current research has provided both theoretical and experimental evidence for magnetoelectric coupling in CCPS, highlighting the critical function of spin–orbit coupling in coupling electric dipoles and spins^40,41,43^. This interaction implies that magnetic fields can cause electric polarization and that electric fields can alter magnetic ordering. This results in a differential effective refractive index for forward and backward propagating light, thus enabling a nonreciprocal transmission spectrum essential for optical isolator functionality. Additional discussions on the magnetic properties of CCPS, including magnetic order under various temperature and magnetic field conditions, are presented in Supplementary Note 3 and Figs.S12, S13.

At a wavelength of 1550 nm, the device demonstrated an insertion loss ranging from 0.15 to 1.8 dB and a high isolation ratio of 28 dB, reflecting its competitive performance among monolithic magneto-optical devices^3,4,14,24,52^. Here, the measured insertion loss is the result of the difference between the baseline transmission (off-resonance) and the transmission at the operating wavelengths for the forward-propagating mode (CCW) (see Fig. S15d)^4^. Note that the coupling loss between the fiber-waveguide (~12 dB per facet) is external to the device’s intrinsic properties and was subtracted from the total loss. The low insertion loss reflects the minimal internal losses due to the integration of CCPS with the silicon photonic ring resonator and the efficient design of the device in its non-reciprocal state.

Further, the resonant peak positions, recorded in both forward and backward directions across repeated tests, showed a consistent nonreciprocal resonant peak shift of (0.21 ± 0.01) nm at 40 mT. Further details of these findings are provided in Supplementary Note 4, which includes comparative analyses of at least 10 tested devices with varying average thicknesses and interaction lengths, along with evaluations of their performance. The quality factor and extinction ratio of the pre-fabricated rings are important determinants of the isolation ratio, with observable effects on both the isolation bandwidth and insertion loss. Refinements in these parameters may lead to improvements in overall device performance.

We also investigated the impact of varying magnetic field strengths on the resonance detuning in the device. As illustrated in Fig. 4b, the device exhibited a maximum shift of approximately 0.4 nm at an upper limit of 55 mT, constrained by our experimental setup. Given the narrowband nature of the microring resonator (MR), any resonance splitting exceeding 0.1 nm facilitates isolation equivalent to the MR’s full extinction ratio^18^. This was observed in our experiments, supporting a 50 GHz optical bandwidth operation. These results conclusively demonstrate the nonreciprocal light propagation in the hybrid system for the TE mode, eliminating the need for polarization rotators as previously implemented in devices that utilize magneto-optic materials^3,59^.

**Fig. 5:**
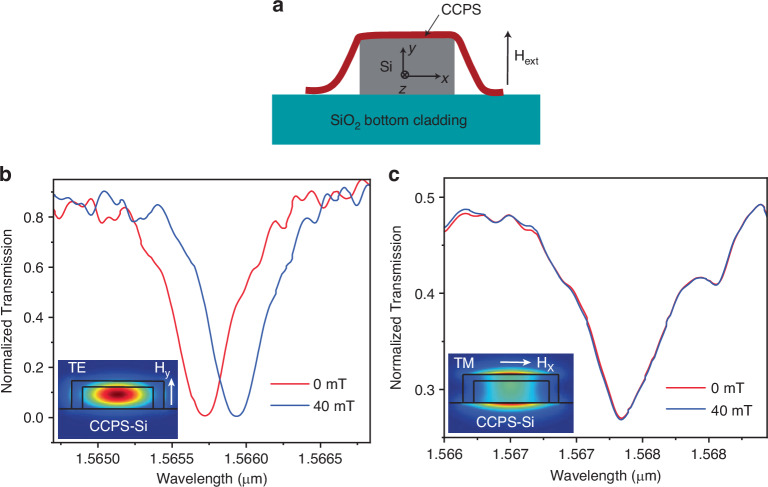
Polarization-dependent characterization of the Si/CCPS hybrid device. **a** Schematic representation of the integrated CCPS/Si device with a magnetic field applied perpendicular to the direction of light propagation. **b** Normalized transmission spectra for the TE mode at magnetic fields of 0 and 40 mT, illustrating resonance detuning. **c** Normalized transmission spectra for TM mode at 0 and 40 mT, demonstrating insensitivity to the magnetic field as evidenced by the unshifted peak positions, the *y*-axis values have been scaled up by a factor of two to improve data visibility. Insets in (**b**) and (**c**) show the electric field profile in the hybrid CCPS/Si device, aligned with the direction of the magnetic field

Further insights are provided by the polarization-dependent measurements, illustrated in Fig. 5a, b. The TE mode exhibits sensitivity to the magnetic field; this is attributed to the alignment of its magnetic field component (*H*_*y*_) with the external field, as shown in Fig. 5a. Conversely, the TM mode, primarily characterized by *H*_*x*_, remains unaffected, due to its perpendicular orientation to the magnetic field. These results demonstrate that the directional and polarization-dependent behavior of the CCPS-integrated device is governed by magneto-optic interactions, highlighting the critical role of magnetic alignment in the tuning of optical properties. Additionally, the observed nonreciprocal light propagation in a CCPS-integrated micro-ring resonator arises solely from magneto-optic effects, with no contribution from thermal effects.

**Fig. 6:**
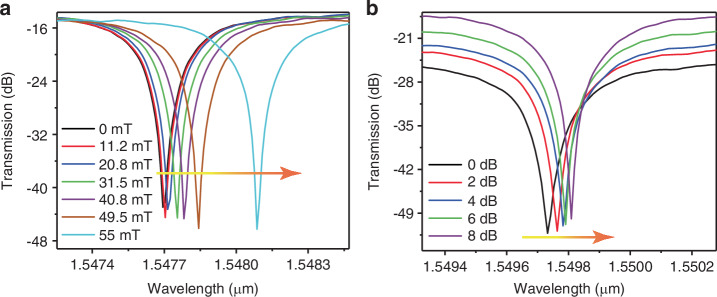
Magneto-optic phase shift (ΔΦ) characteristics of the hybrid CCPS/Si micro-ring resonator (MRR). **a** Transmission spectra for the TE mode with a constant input laser power of 10 dBm, launched from the left (fixed direction), as the magnetic field varies from 0 to 55 mT. **b** Transmission spectra for the TE mode under a steady magnetic field of 50 mT, with input laser power adjusted from 0 to 8 dBm, launched from the left

To further investigate the magneto-optic interaction in our hybrid CCPS-Si waveguide system, we conducted complementary experiments to explore the phase modulation capabilities of the device. While the primary focus of this work is on the nonreciprocal response, these additional experiments provide deeper insight into the underlying magneto-optic effects governing the observed behavior. In contrast to the nonreciprocal measurements, where light propagation direction and magnetic field polarity were alternated to evaluate resonance splitting (Δ*λ* = *λ*_CW_−*λ*_CCW_), the phase modulation experiments were designed to isolate the magneto-optic phase shift under a fixed light injection direction (ΔΦ). Specifically, the light was launched from a fixed port while the magnetic field was incrementally increased in a single polarity (this experimental setup was used in Figs. 6, S16, and S17). This approach was applied in two separate measurements: first, with light propagating from the left port, and subsequently, with light propagating from the right port. The results allow for a direct assessment of the magnetically induced phase shift without introducing directional nonreciprocity, offering a complementary perspective on the device’s potential for active phase tuning. Importantly, while these phase modulation measurements are fundamentally distinct from the nonreciprocal operation, they remain inherently connected through the magneto-optic interaction between CCPS and the guided optical mode. Further details on the distinctions between the nonreciprocal testing (Δ*λ*) and phase shift tuning experimental (ΔΦ) setups can be found in Table S1.

Figure S16 presents the measured phase shift, revealing an asymmetric response depending on the propagation direction. The phase shift induced when light is launched from the left differs in magnitude from that observed when launched from the right under the same magnetic field conditions. This asymmetry represents a notable deviation from the symmetric behavior expected under purely thermal effects, providing further evidence of the magneto-optic influence in the device^60^.

Figure 6a displays the transmission spectra for the TE mode at a fixed input laser power of 10 dBm, with light injected from the left port. The phase shift (ΔΦ), measured across a magnetic field range from 0 to 55 mT, exhibits a nonlinear response, as further detailed in the supporting information (Fig. S17). This nonlinear behavior arises from the magneto-optic interaction between the optical mode and the magnetized CCPS layer, which may include contributions from the material’s intrinsic nonlinear magnetization response. Various factors, such as strain-induced birefringence and magnetoelectric coupling, could collectively contribute to the observed phase shift. Additional details can be found in Supplementary Note 5.

In addition, Fig. 6b shows the TE mode transmission spectra under a constant magnetic field of 50 mT, with input laser power varied from 0 to 8 dBm. Notably, the observed phase shift demonstrates a power-dependent behavior, which contrasts with the typical characteristics of the Faraday effect that is generally independent of optical power.

These findings suggest that the observed magneto-optic interaction in our device is not solely governed by the Faraday effect but may instead be influenced by alternative mechanisms, such as the magneto-optical Kerr effect (MOKE). Furthermore, the unique magnetoelectric properties of CCPS, which facilitate strong spin–orbit coupling, may also contribute to the observed phase shift behavior. Collectively, these effects highlight the complex interplay of magneto-optic phenomena in our system and underscore the distinct advantages of 2D materials for integrated photonics applications^61^.

**Fig. 7:**
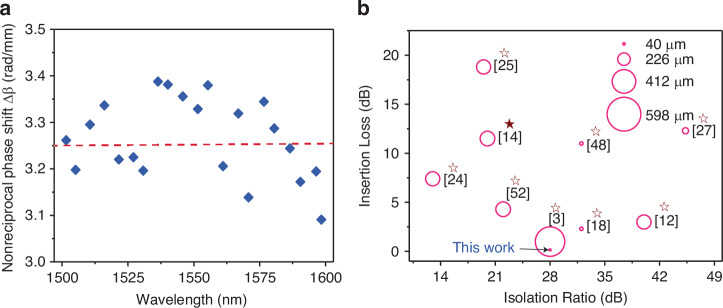
Performance of the nonreciprocal hybrid CCPS/Si- MRR device. **a** Measured nonreciprocal phase shift (*Δβ*) across a range of wavelengths. **b** Comparative analysis of research on magneto-optic (MO) materials integrated into ring resonators near the C-Band, focusing on isolation ratio, insertion loss, and MO material integration length, in this study, a maximum magnetic field of 55 mT was applied. Note that the hollow star represents a ring resonator, while the filled star denotes an MZI structure

Dispersion is another critical factor in determining the performance of nonreciprocal photonic devices. Therefore, we evaluated the dispersion characteristics of the nonreciprocal phase shift (Δ*β*) over the wavelengths from 1500 to 1600 nm, as depicted in Fig. 7a. The dispersion exhibited by Δ*β* is relatively low across the measured wavelength range. It maintained an approximate value of 3.25 rad/mm, which suggests a potential for broader operational bandwidth. This modest level of dispersion could help reduce the wavelength dependency and dispersion limitations often encountered in conventional nonreciprocal devices^13^.

Furthermore, our measurements indicate a consistent nonreciprocal response extending from 1500 to 1600 nm, and from 1280 to 1360 nm, as referenced in Fig. S18. The observed operational bandwidths are 100 and 80 nm, respectively, in these ranges. These findings underscore the enhanced performance and versatility of our hybrid CCPS/Si devices, highlighting their potential for broader applications in photonic systems.

Figure 7b displays a bubble chart comparing magneto-optic (MO) materials integrated into ring resonators near the C-Band. It outlines the isolation ratio, insertion loss, and MO material integration length across different studies. Notably, our hybrid CCPS/Si device features a compact interaction length of 22–55 µm and a thickness range of 39–62 nm, minimizing the device’s footprint while maintaining low insertion losses and a high isolation ratio.

Our device operates in the TE mode consistent with the main polarization used in silicon photonics integrated circuits^62^. It is in contrast with other studies that primarily target the TM mode and often require additional components, such as polarization rotators, to achieve nonreciprocal TE mode responses^3,59^. These components typically contribute to higher insertion losses. The direct TE mode operation of our CCPS/Si device avoids these complexities, offering a simpler and potentially more efficient solution for photonic circuits, particularly in applications where future integrated lasers predominantly operate in TE modes. This functionality aligns with the practical needs of advanced optical systems.

## Discussion

The investigation into the magneto-optic response of multilayer CCPS integrated into silicon photonics reveals notable advancements in integrated non-reciprocal optical devices at near-infrared wavelengths. The intralayer ferromagnetic ordering within CCPS, characterized by an easy-plane magnetocrystalline anisotropy, significantly influences the magneto-optical properties and induces modal asymmetry under magnetic fields. This anisotropy is key for the non-reciprocal phenomena observed, making the CCPS/SiPh devices effective in balancing low optical losses with strong light–matter interaction.

The device exhibits a low insertion loss ranging from 0.15 to 1.8 dB and an isolation ratio of 28 dB at a wavelength of 1550 nm. These metrics demonstrate the device’s functional efficiency and competitive performance relative to monolithic alternatives. The design favors the transverse electric (TE) mode commonly used in silicon photonics circuits, avoiding the need for extra components like polarization rotators, which typically add to losses and complexity.

Additionally, the device’s compact dimensions, with a 22–55 µm interaction length and 39–62 nm thickness, help reduce its footprint while maintaining functionality. The observed resonance splitting of 0.4 nm at an applied magnetic field, supporting a 50 GHz optical bandwidth, along with low dispersion allowing for operational bandwidths up to 100 nm, showcases the device’s capability for broader applications.

It is important to note that the optical bandwidth is determined by a balance between the acceptable isolation ratio and insertion loss, rather than by resonance splitting alone. While resonance splitting provides a measure of the device’s ability to achieve nonreciprocal behavior, the practical operational bandwidth is more dependent on the tolerable degradation in isolation ratio and insertion loss.

In our study, the reported resonance splitting indicates the range within which nonreciprocal effects are most pronounced. However, we recognize that the usable optical bandwidth is governed by the point at which the isolation ratio remains sufficiently high and the insertion loss remains low enough for effective performance in high-speed applications. Typically, optical isolators connected to lasers are employed in low-bandwidth applications, where high-speed modulation is not required. Their primary function is to prevent back reflections from reaching the laser, ensuring stable operation, especially in single-frequency lasers.

Our results demonstrate the potential of hybrid CCPS/Si devices as efficient and compact optical isolators in the short-wave infrared range, crucial for minimizing back-reflection in advanced optical systems. This work advances the development of highly integrated nonreciprocal photonic devices for future optical networks.

## Materials and methods

### Materials supply

Bulk CuCrP_2_S_6_ crystals were sourced from HQ Graphene (https://www.hqgraphene.com/).

### Scanning electron microscopy (SEM) and X-ray dispersive spectroscopy (EDX)

Photonic chips were mounted on SEM stubs using carbon tape and imaged under high vacuum mode with a Quanta 450 field emission scanning electron microscope (FEI) at an electron energy of 10 kV. For compositional analysis, EDX mapping was performed on a CCPS bulk crystal also mounted on SEM stubs with carbon tape. EDX spectra were acquired at an electron energy of 30 kV.

### Atomic force microscopy (AFM)

AFM measurements were conducted using a WITec AFM module integrated with a research-grade optical microscope, operating in tapping mode. The cantilever tip used was a Scanasyst-air with a tip radius of 7 nm, a force constant of 0.2 N m^−1^, and a resonance frequency of 14 kHz.

### Transmission electron microscopy (TEM)

High-resolution analytical scanning/transmission electron microscopy (S/TEM) was performed using an FEI Talos F200X operating at 200 keV. This instrument combines high-resolution S/TEM imaging with a four-quadrant energy-dispersive X-ray spectrometer (EDS) for detailed elemental and compositional mapping. The sample was prepared as a thin lamella, where e-carbon and ion carbon layers were applied as protective coatings to ensure the integrity of the lamella during imaging.

### X-ray photoelectron spectroscopy (XPS)

The composition of the CCPS was analyzed using X-ray photoelectron spectroscopy (XPS) on a Nexsa G2 XPS system (Thermo Fisher Scientific). This system incorporates SnapMap™ imaging, enabling precise alignment of small X-ray spots with bond pads for accurate surface chemical analysis. Measurements were performed using a monochromatic Al Kα X-ray source.

### Magnetic force microscopy (MFM)

Magnetic force microscopy (MFM) measurements were conducted using an NX10 atomic force microscope (AFM) system equipped with a magnetic field generator (Park Systems). The magnetic field generator applied an in-plane magnetic field with a maximum strength of ~335 G to the sample. The MFM mode was employed with a lift height of 6 nm. The samples consisted of CCPS 2D material deposited on a SiO_2_/Si substrate. The flakes exhibited varying sizes and thicknesses, and two relatively thin flakes were selected for detailed measurements under different applied magnetic fields.

### Mode analysis and FDTD simulation

The electric field profile within the silicon waveguide was determined using the eigenmode solver in MODE Solutions, part of Lumerical’s Device Multiphysics Simulation Suite.

### Spectroscopic imaging ellipsometer

The optical properties of multilayer CuCrPS were assessed using an Imaging Ellipsometry system from Accurion (https://accurion.com/company). This system combines optical microscopy with ellipsometry, enabling spatially resolved measurements of layer thickness and refractive index. The technique is highly sensitive to ultrathin films, capable of analyzing structures from sub-nanometer single layers to multilayers several microns in thickness. Imaging Ellipsometry provides layer thickness measurements with a spatial resolution of up to 1 µm. The ellipsometric parameters Psi (ψ) and Delta (Δ) were evaluated and fitted using EP4 model software.

### Stamping of CCPS on the photonic chip

Multilayered flakes were exfoliated using Scotch tape and subsequently transferred onto a PDMS substrate. Employing a precise dry transfer method, selected CCPS flakes were positioned onto the Si waveguides. Detailed information on the deterministic transfer process is provided in the supplementary material.

### Optical characterization

The optical transmission was measured by edge coupling light into the device structure using lensed fibers connected to a tunable laser in the short-wave infrared (SWIR) band (Keysight 8164B Lightwave Measurement System). The output light from the devices was collected with an output lensed fiber and detected by a power meter. Polarization of the light (TE/TM) was calibrated using reference rings fabricated on the same chip with identical geometries. Calibration of output optical power intensities was performed with a standard photodiode power sensor prior to device testing. All optical measurements were performed in air at room temperature. The chip was mounted on an electromagnet stage, which was controlled by a DC power supply. Additional details about the experimental setup can be found in Supplementary Note 2.

## Supplementary information

Supplementary Information

## Data Availability

The data that support the findings of this study are available from the corresponding author, upon reasonable request.
