# Supplementary material for: Non-reciprocal response in silicon photonic resonators integrated with 2D CuCrP2S6 at short-wave infrared
Source: Light Sci Appl. 2025 Apr 9;14:157. doi: 10.1038/s41377-025-01826-w (PMC11982241; doi:10.1038/s41377-025-01826-w)
Supplement: Supplementary file 1 — Supplementary Information [file 41377_2025_1826_MOESM1_ESM.docx]

**Supplementary Information for**

**Non-Reciprocal Response in Silicon Photonic Resonators Integrated with 2D CuCrP_2_S_6_ at Short-Wave Infrared**

Ghada Dushaq^1^*, Solomon Serunjogi^1^, Srinivasa R. Tamalampudi^1^, and Mahmoud Rasras^1,2^*

^1^ Department of Electrical and Computer Engineering, New York University Abu Dhabi, P.O. Box 129188, Abu Dhabi, United Arab Emirates

^2^ NYU Tandon School of Engineering, New York University, New York, USA

**This file includes:**

**Figure S1.** Structural properties of CCPS

**Figure S2.** Energy dispersive X-ray (EDX) analysis of a CCPS sample

**Figure S3.** X-ray photoelectron spectroscopy (XPS) data

**Figure S4.** Transmission electron microscopy imaging

**Figure S5.** Passive optical properties of bare Si MRR

Supplement note 1 **Breaking X-axis symmetry, modal analysis and optical loss analysis in ring resonators with multilayer CCPS integration**

**Figure S6.** Schematic representation and refractive index distribution of the hybrid CCPS-Si waveguide structure

**Figure S7**. Simulated mode analysis of the hybrid CCPS-Si waveguide structure under forward light propagation

**Figure S8.** Simulated mode analysis of the hybrid CCPS-Si waveguide structure under backward light propagation

**Figure S9.** Passive transmission spectra of the mirroring resonator around 1550 nm with fitting

Supplement note 2 **Electromagnet and experimental setup**

**Figure S10.** Experimental setup for magneto-optic testing

**Figure S11.** Transmission spectra of the CW and CCW light propagation in a bare Si MRR under a 40 mT applied magnetic field

Supplement note 3 **Magnetic properties and magnetoelectric coupling in CuCrP₂S₆ (CCPS)**

**Figure S12.** Magnetic properties of CCPS

**Figure S13.** Magnetic force microscopy (MFM) analysis of CCPS nanoflakes

Supplement note 4 **Comparative analysis, material integration, and device testing**

**Figure S14.** Photonic chip design and fabrication

**Figure S15.** Comparative analysis

Supplement note 5 **Magneto-optic phase shifter (ΔΦ)**

**Figure S16.** Resonance shift as a function of magnetic field

**Figure S17.** Magneto-optic characteristics of the hybrid CCPS/Si micro-ring resonator (MRR).

**Table S1 Summary of experimental distinctions between nonreciprocal and magneto-optic phase shift measurements in the CCPS-Si hybrid system**

**Figure S18.** Transmission spectra of CW and CCW light propagation of 1310 centered laser light under 40 mT magnetic field

**References**

**Structural properties of CCPS**

The layered Van der Waals material CuCrP₂S₆ (CCPS) is part of the transition metal thio/selenophosphates (TPS) family, characterized by a monoclinic crystal structure (Pc space group)^1,2^. Figure S1a presents a 3D schematic (bc plane) of the material's structure. Each monolayer consists of a sulfur framework with octahedral cages occupied by Cu and Cr ions, as well as P-P pairs. Cu ions alternately occupy upper (Cu1) and lower (Cu2) positions, leading to an antiferroelectric (AFE) state at low temperatures. The crystal structure forms triangular networks composed of quasi-trigonal CuS₃, octahedral CrS₆, and P₂S₆ units. Cr ions and P-P pairs are almost centrally located within a layer, while Cu ions are slightly off-centered^1,3,4^. Figures S1b and S1c present the AFM image scan and the cross-sectional thickness measurements of a stair-like CCPS flake.**Top of Form**

**
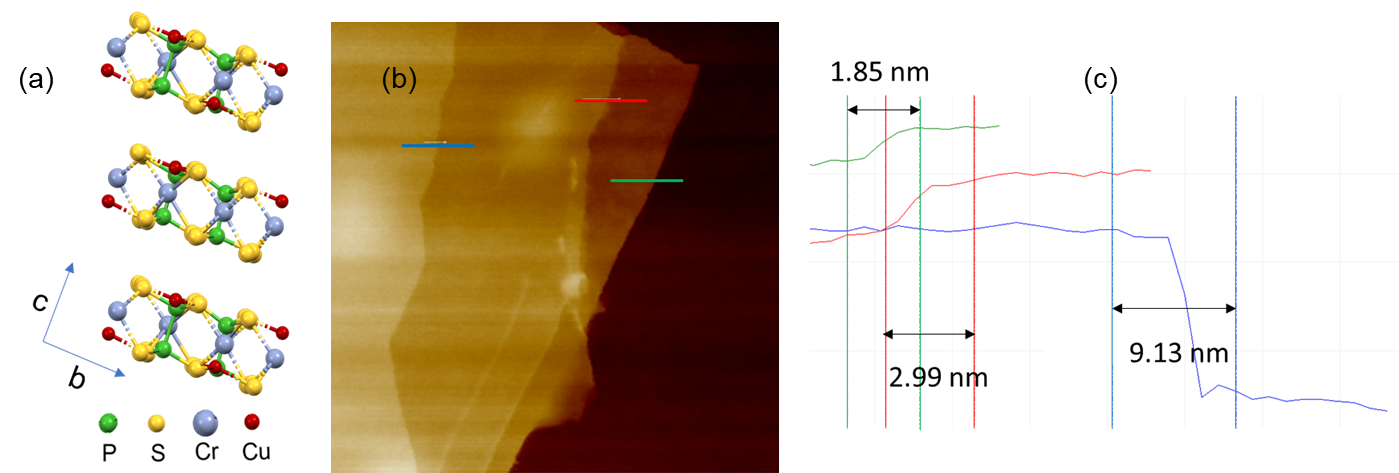
**

**Figure S1.** **Structural properties of CCPS: (a)** 3D representation (ball and stick model) of the *bc* plane. **(b)** AFM image scan of a stair-like CCPS flake. **(c)** Cross-sectional thickness measurements, with green, red, and blue step heights corresponding to the colored solid lines in the AFM scan.

**
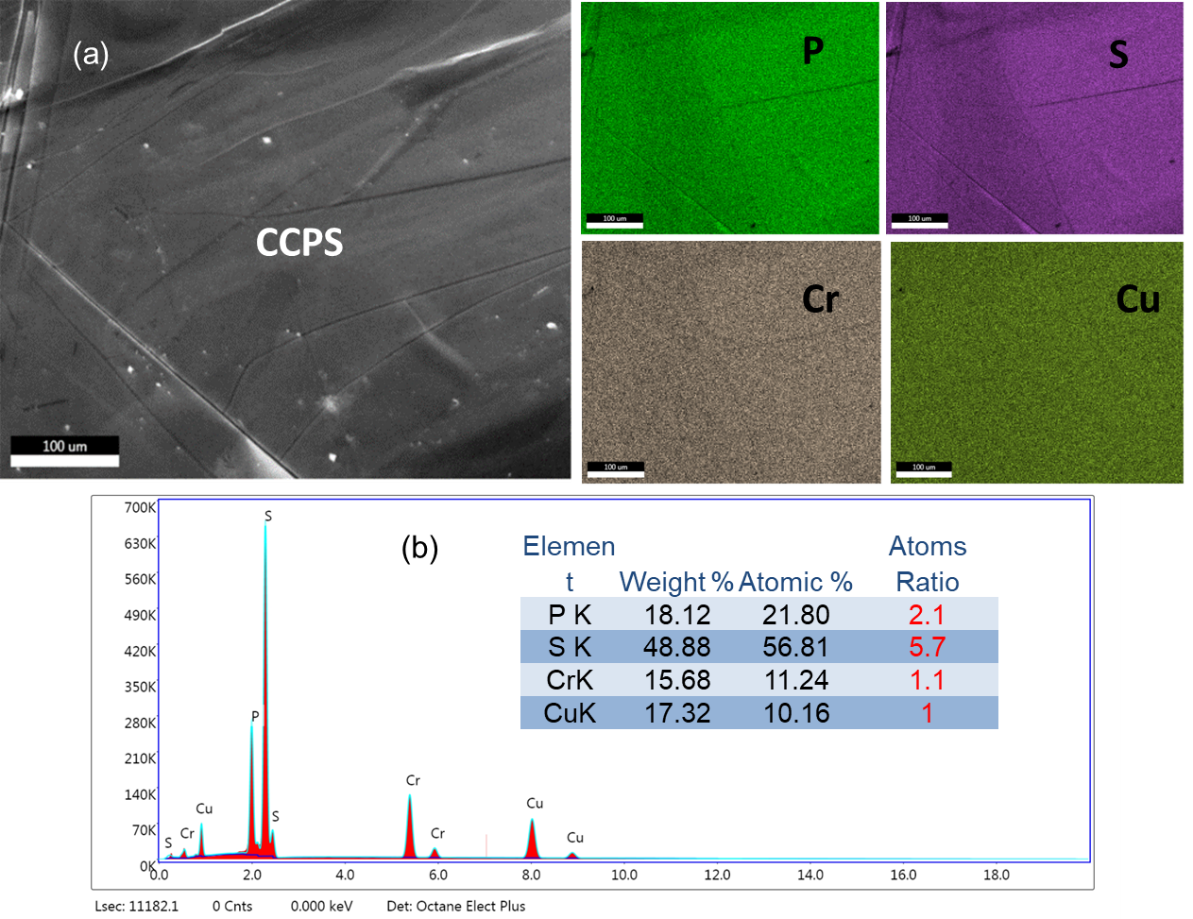
**

**Figure S2.** **Energy dispersive X-ray (EDX) analysis of a CCPS sample using Scanning Electron Microscopy (SEM): (a)** SEM image of the analyzed area with quantitative EDX mapping of CCPS. **(b)** EDX spectra displaying the detected elements, accompanied by a table presenting the atomic and weight percentage composition of CuCrP₂S₆. The scale bar in (a) represents 100 µm. The observed ratio of 1:1.1:2.1:5.7 closely matches the expected stoichiometric composition of CCPS (1:1:2:6).

***
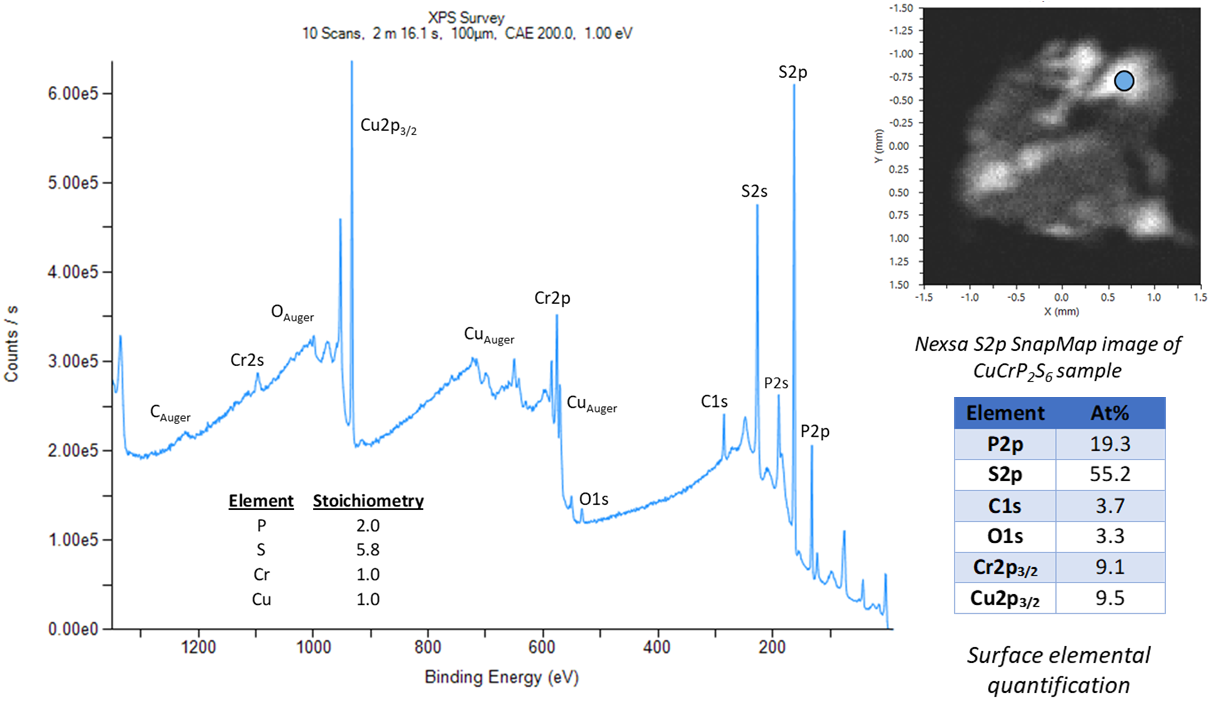
***

**Figure S3. X-ray photoelectron spectroscopy (XPS) data acquired using the Thermo Scientific Nexsa G2:** The spectrum is from a selected location on the SnapMap™ image (blue circle) and shows the stoichiometric composition of CCPS (1:1:2:5.8), closely matching both the EDX data and the expected stoichiometry.

**.**

**
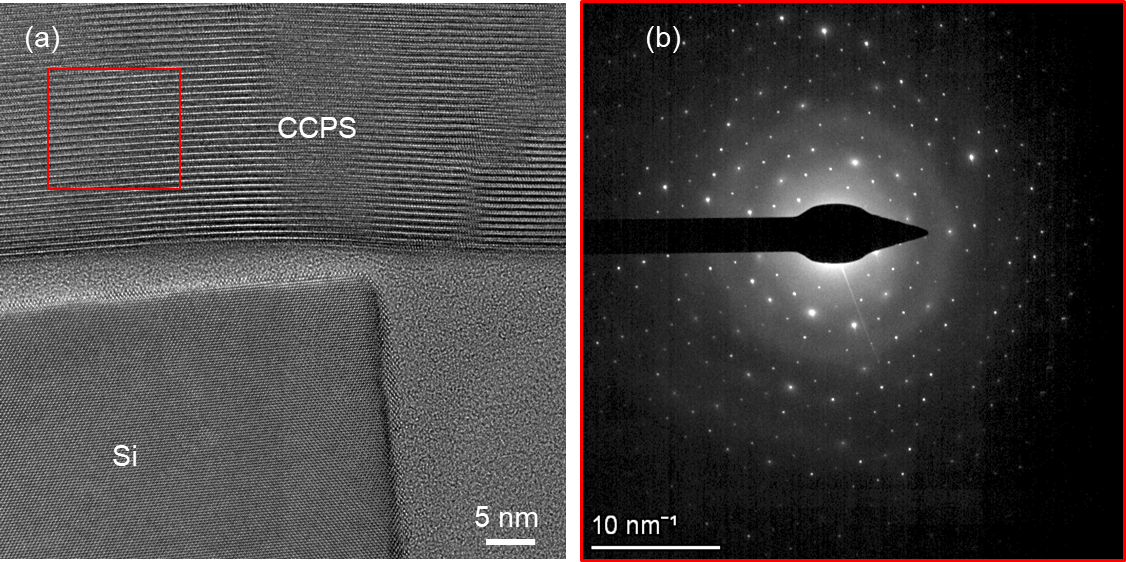
**

**Figure S4.** **Transmission electron microscopy imaging: (a)** High-resolution image of the Si waveguide and integrated CCPS interface, highlighting its crystallinity. **(b)** Selected area diffraction pattern (SADP) recorded from the red square region in (a).

**
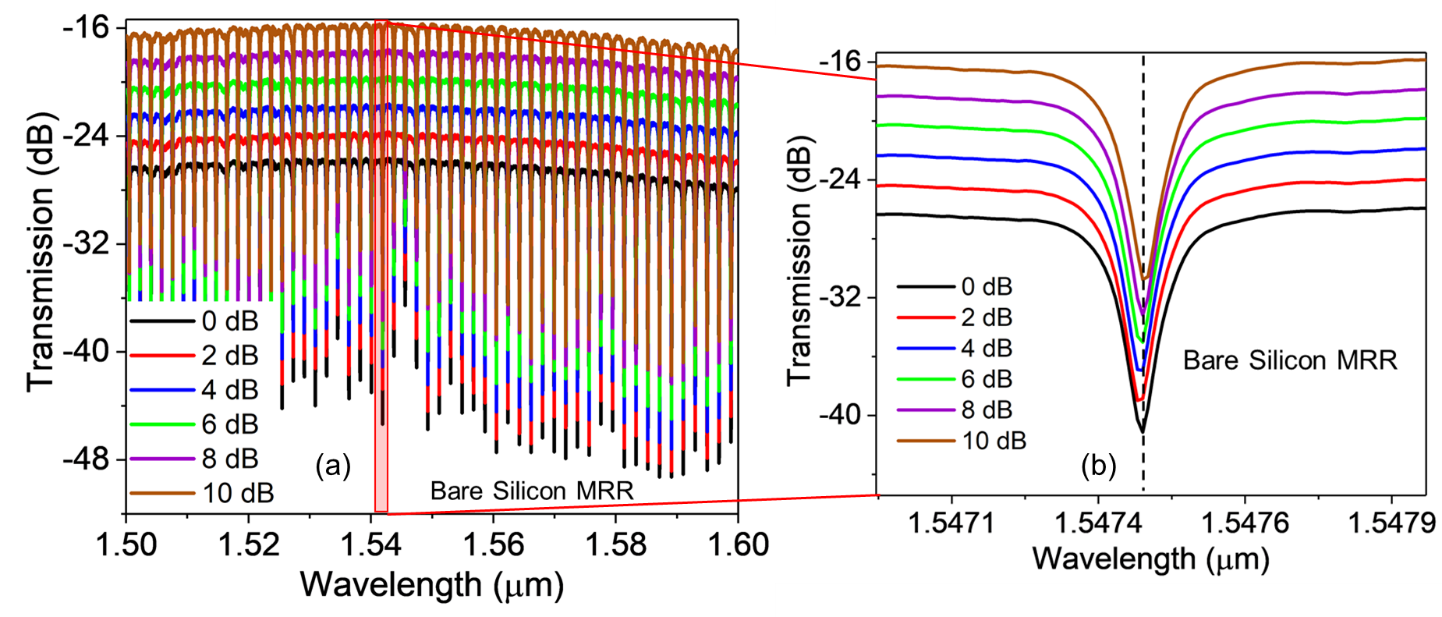
**

**Figure S5. Passive optical properties of bare Si MRR. (a)** transmission spectra spanning 1500 nm to 1600 nm of the Si MRR at varying input optical powers. **(b)** detailed view of the resonance peak, illustrating minimal thermal dissipation from light propagation within the Si MRR.

**Supplement note 1**

**Breaking X-axis symmetry in waveguide**

For a perfectly X-symmetric waveguide, the electric field Ex(x,y) for the TE mode is symmetric, and its spatial derivative ∂xEx(x,y) is antisymmetric, leading to a zero integral in equation (2). This symmetry typically prevents NRPS in the TE mode, which is why NRPS is commonly observed in the TM mode for such structures.

However, in our device, several key factors break this symmetry, allowing for a nonreciprocal phase shift in the TE mode:

1. Waveguide Asymmetry: The integration of CCPS onto the waveguide introduce asymmetry due to irregularities in flake deposition and strain effects. These factors can cause variations in the refractive index distribution across the waveguide cross-section, breaking the X-symmetry that typically inhibits NRPS in the TE mode. Specifically, the non-uniform deposition of the CCPS flake can impact mode propagation and the electric field distribution, enabling nonreciprocal phase shifts in modes that would otherwise exhibit symmetry, such as the TE mode.
2. Strain-Induced Refractive Index Changes: Our waveguide structure is un-cladded and non-planar, which induce strain in the CCPS flake upon deposition. Strain-induced refractive index variations are well-documented for 2D materials^5–7^, and this strain can generate asymmetry in the waveguide's effective refractive index profile. Such changes affect both the optical properties of the waveguide and the CCPS layer, contributing further to symmetry breaking and allowing for the observed nonreciprocal phase shift in the TE mode.
3. Asymmetric Coverage of the Waveguide Sidewalls: In our air-cladded, striped waveguide, the deterministic transfer of the CCPS layer results in asymmetric coverage of the waveguide's sidewalls. This X-asymmetry is a known contributor to nonreciprocal behavior, similar to previous demonstrations where iron garnet was deposited asymmetrically on the sidewalls of waveguides to enable direct TE-mode nonreciprocal response^8^. The asymmetric shape of the CCPS coverage on the sidewalls plays a significant role in breaking the symmetry and enabling nonreciprocal phase shift in the TE mode.

The TE mode is indeed excited in our experiments which is confirmed through comprehensive modal analysis, which matches the free spectral range (FSR) both theoretically and experimentally, as well as through polarization tests conducted on the same chip. Therefore, the observed nonreciprocal phase shift is conclusively associated with the TE mode.

Additionally, 2D materials can enable TE-mode NRPS through optical symmetry-breaking mechanisms, even in the absence of structural asymmetry. For example, recent studies on graphene and graphene oxide demonstrate that uniform coverage can result in nonreciprocity for the TE mode due to intrinsic properties like photonic spin-orbit interaction (PSOI) or photo-thermal effects^9,10^. While our device leverages structural asymmetry introduced by the non-uniform CCPS coverage and strain profile, it also exploits material-specific properties, such as magnetoelectric coupling and strain-induced birefringence, to enhance the nonreciprocal response. These properties provide additional degrees of freedom for breaking symmetry and enabling TE-mode NRPS, distinguishing our system from traditional designs based on uniform 3D magneto-optic films. Further details regarding the refractive index profile, mode field distributions, and non-zero Δβ for TE modes are provided in the modal analysis section.

**Modal analysis and electromagnet field propagation of hybrid CCPS/Si**

Our device integrates a multiferroic 2D material (CCPS) with non-uniform coverage, strain effects, and magnetoelectric properties, which collectively break the X-symmetry of the waveguide. This symmetry breaking is critical for enabling NRPS in the TE mode, as it disrupts the otherwise symmetric electric field distribution along the X-axis. The TE mode, with its predominantly horizontal electric field component, is particularly sensitive to in-plane refractive index variations introduced by the non-uniform CCPS layer. In addition to non-uniform coverage, the integration of CCPS on top of the fully etched waveguide introduces non-uniform strain due to the aspect ratio of the waveguide and the nanophotonic nature of the interaction. This strain contributes to an asymmetric refractive index profile, which further influences the optical mode and enhances the symmetry-breaking effect. Importantly, in integrated photonic systems like Si-photonics, where optical modes are highly confined, even small perturbations such as non-uniform strain, flake coverage, or thickness variations can significantly alter the mode profile and its interaction with the material. This high field confinement amplifies their impact, making them critical for breaking symmetry and enabling nonreciprocal effects.

To validate this mechanism, we performed simulations using Lumerical Mode Solutions and electromagnet beam propagation incorporating the asymmetric refractive index profile resulting from the non-uniform coverage and strain in the CCPS layer. It is worth mentioning that the TEM cross-section revealed a clear gap of approximately 0.5 µm between the CCPS layer and the waveguide sidewalls. This gap is dependent on the initial CCPS thickness, and the stamping process accuracy. Based on this information, we performed mode simulations, considering that the CCPS does not cover the sidewalls and that the 0.5 µm gap is sufficiently large to neglect any field interaction. In these simulations, the CCPS was placed only on top of the waveguide.

Figure S6a shows the measured optical parameters of CCPS obtained using a high-resolution ellipsometer. These parameters were subsequently incorporated into Lumerical software to simulate the hybrid CCPS/Si system. The 3D model of the device geometry, presented in Figure S6b, reveals the non-uniform coverage of the CCPS layer across the silicon waveguide, resulting in strain-induced variations in the refractive index. To further elucidate these effects, cross-sectional refractive index profiles were extracted from two distinct positions along the waveguide, denoted as "Cross section from right" and "Cross section from left" (see Fig. S6c). These profiles confirm the presence of spatially varying optical properties within the hybrid waveguide system, which play a critical role in the observed nonreciprocal phase shift.

**
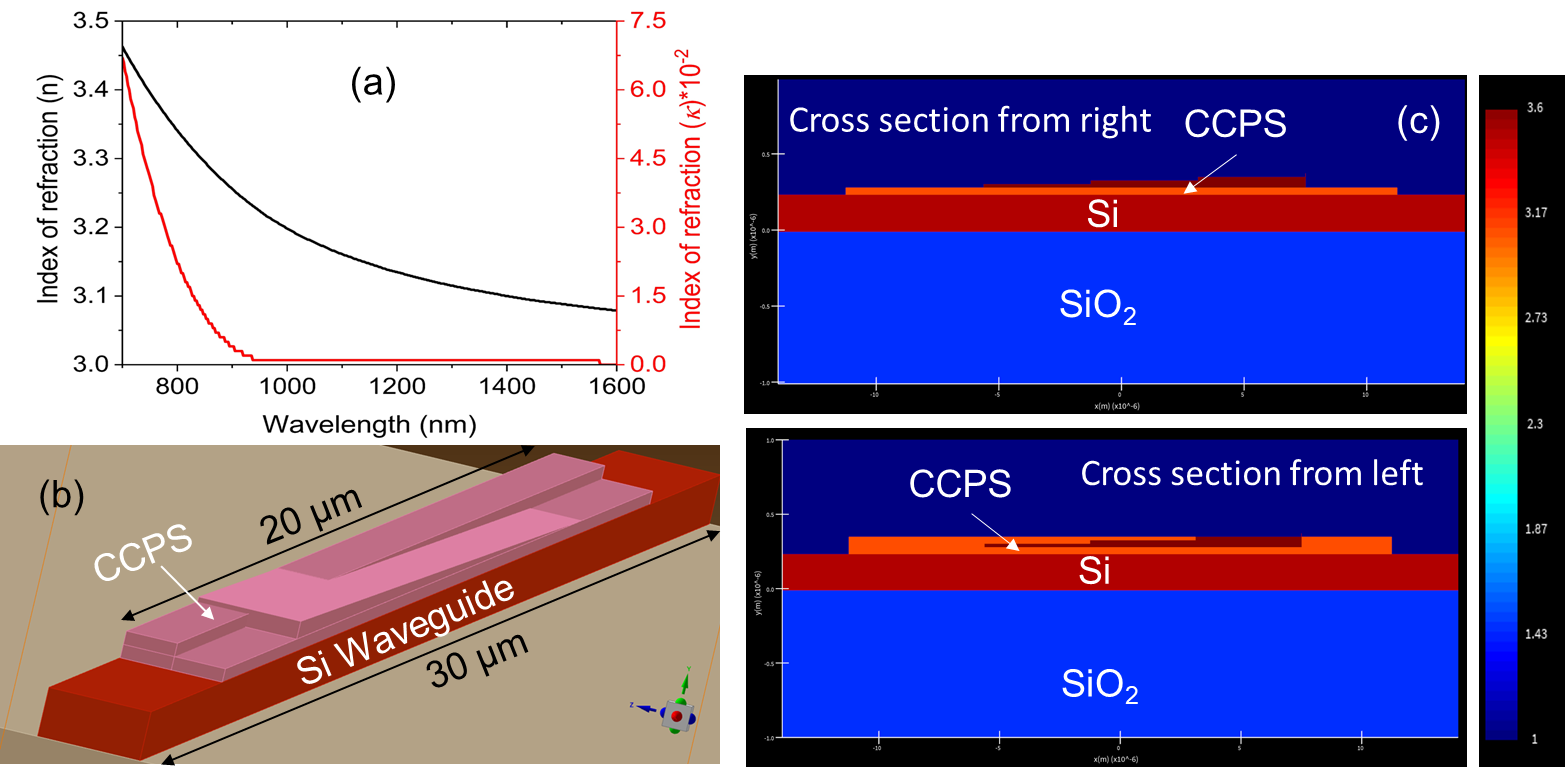
**

**Figure S6.** **Schematic representation and refractive index distribution of the hybrid CCPS-Si waveguide structure.** **(a)** Optical parameters, refractive index (n) and extinction coefficient ($\kappa$), measured via high-resolution ellipsometry **(b)** Constructed 3D model illustrating the non-uniform CCPS coverage on the silicon waveguide, resulting in strain-induced refractive index gradients. **(c)** Cross-sectional views of the refractive index profile at two different positions along the waveguide, denoted as "Cross section from right" and "Cross section from left," highlighting the spatial variations in refractive index due to the non-uniform CCPS integration. The color scale represents the refractive index distribution.

The optical parameters of CCPS with varying thicknesses were extracted from high-resolution ellipsometry measurements and incorporated into the Lumerical simulations. Strain was modeled as a trapezoidal perturbation to the refractive index (Δn).

Furthermore, we analyzed the propagation of light along the waveguide in both forward and backward directions by reversing the input light source. Figure S7 presents the forward propagation case, where optical mode profiles were monitored at eight equidistant positions along the waveguide [light source is at (1)]. Figure S8 illustrates the corresponding results for backward propagation, where the same monitoring positions were used with light launched from the other direction [equivalent to have light source at (8) in Fig.S7]. In each case, the effective refractive index (neff​) values and mode profiles were extracted at each monitor position. The results clearly demonstrate that the non-uniform CCPS coverage induces significant asymmetry, effectively breaking the X-symmetry of the TE mode and leading to a non-zero contribution to the nonreciprocal phase shift (NRPS) integral, as described in equation (2) in the main manuscript. The calculated propagation constant difference (Δβ) for the TE mode exhibits a strong correlation with the degree of asymmetry, in agreement with our experimental findings. As outlined in the main manuscript (Equations 3 and 4), the observed changes in effective index (Δneff​) are directly proportional to the variations in Δβ. It is important to note that the simulations incorporate a significant degree of asymmetry, resulting in a calculated Δneff​ (neff(b)-neff(f)) between forward (f) and backward (b) direction ranging from 1×10^−3^ to 0.1. In contrast, our experimental measurements, as detailed in Supplementary Note 4, indicate that an effective index change of 0.3×10^-3^ to 1×10^−3^ is sufficient to induce a measurable resonance splitting under magnetic field, highlighting the sensitivity of the system to minor asymmetry perturbations.


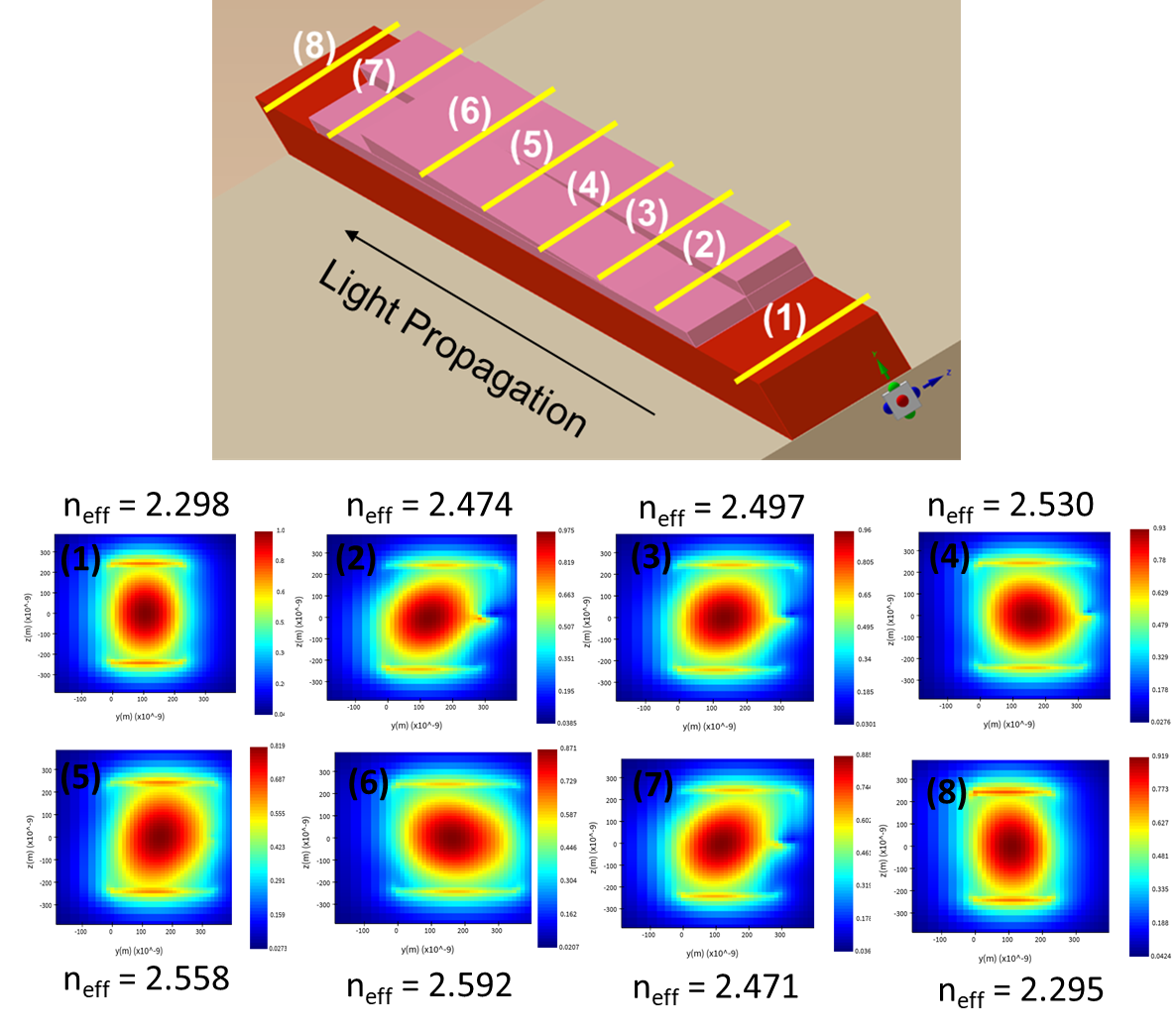


**Figure S7.** **Simulated mode analysis of the hybrid CCPS-Si waveguide structure under forward light propagation.** The top schematic illustrates the device configuration with eight monitoring positions along the waveguide length, marked as (1) to (8). The bottom images depict the corresponding transverse electric (TE) mode profiles at each monitoring position, with their respective effective refractive index (neff​) values. The results highlight the gradual variation in mode distribution due to the non-uniform CCPS coverage and strain-induced index gradients, confirming the asymmetry in the optical field distribution.


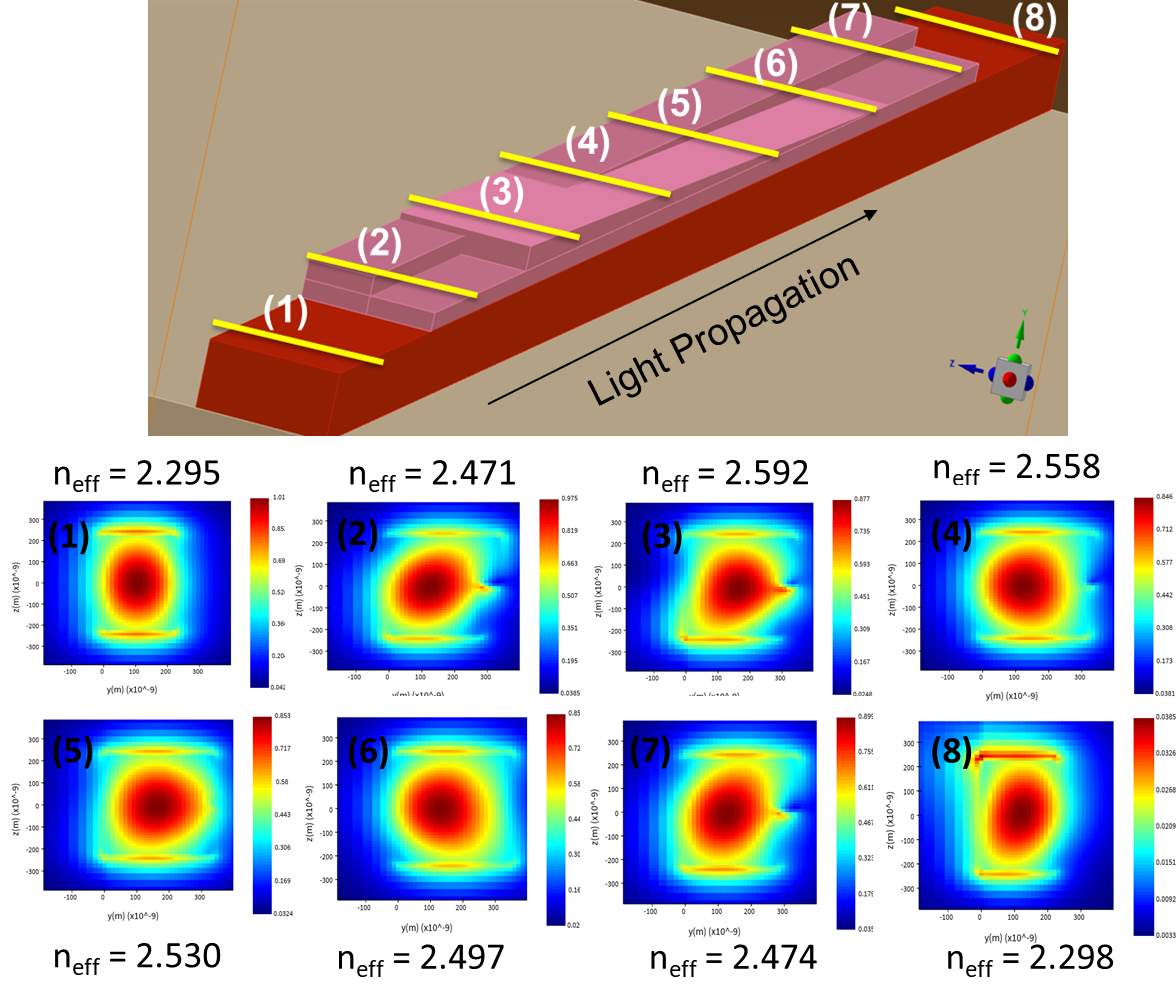


**Figure S8.** **Simulated mode analysis of the hybrid CCPS-Si waveguide structure under backward light propagation.** The top schematic presents the device with eight monitoring positions along the waveguide length, numbered (1) to (8), with the light source injected from the opposite direction to the one shown in Figure RR3. The bottom images show the TE mode profiles at each position, alongside their corresponding effective refractive index (neff) values. **A distinct variation in the mode distribution compared to the forward propagation case further supports the breaking of X-symmetry, leading to a non-zero contribution to the light propagation constant (Δβ).**

**Optical loss analysis in ring resonators with multilayer CCPS integration**

To quantify the change in optical loss per unit length (Δα) due to the integration of multilayer CCPS in the evanescent field region of the ring resonators, we observed the reduction in the ring’s quality factor (Q)^11^. The intrinsic quality factor (Q) of the ring is related to the loss per unit length through the equation^12,13^:

$Q=\frac{10}{ln(10)}\frac{2\pi n_{g}}{\lambda_{0}\alpha}$ (1)

Where 𝜆_0_​ is the resonance wavelength, and 𝑛𝑔 is the group index.

We extracted the loaded quality factor (𝜆_0_/FWHM) from the experimental transmission spectra using Lorentzian fitting (see Figure S7). The intrinsic quality factor (Q_i_) can be determined from the loaded quality factor (Q_l_) using the relationship^14^:

$Q_{i=}\frac{{2Q}_{l}}{1\pm\sqrt{T_{0}}}$ (2)

Where 𝑇_0_​ is the normalized transmitted power at the resonance wavelength.

The device design is optimized for critical coupling conditions at 1550 nm for the TE mode, resulting in an intrinsic quality factor of approximately 2𝑄_𝑙_. In the post-integration of CCPS, losses mainly arise from reflections and scattering at the coupling interface between the passive waveguide (air/Si) and the hybrid region. This is attributed to mode mismatch and flake irregularities.

The change in optical loss (Δα) can be expressed as^12^:

$$\Delta\alpha=\frac{2\pi n_{g}}{\lambda_{0}}\frac{10}{\ln(10)}\left( \frac{1}{Q_{f}}-\frac{1}{Q_{i}} \right) (3)$$

Note: 10/ln (10) is the conversion from linear to logarithmic. 𝑄_𝑓_ is the quality factor of the ring after CCPS integration, and 𝑄_𝑖_ is the quality factor before integration. The group index 𝑛𝑔 (~ 4.2) was obtained from FDTD Lumerical simulations. The quality factors before and after integration were calculated from the FWHM of the resonator’s transmission spectra, as shown in Fig. S9, using Lorentzian fitting. By substituting the measured quality factor values into Eq. (3) and using Eq. (2) to obtain the intrinsic Q, the optical loss due to CCPS can be determined. This value was then normalized to the CCPS interaction length (L_Mo_).

We conducted measurements on both thin (~39 nm) and thick (~127 nm) CCPS flakes. The transmission spectra for these flakes are presented in Fig. S9. For thin flakes, optical losses were found to be negligible, while for thick flakes, losses reached up to 0.018 dB/µm. This significant loss in thick flakes can be attributed to increased light scattering at the input and output regions of the CCPS, caused by the larger step height.

Our comparative analysis of multiple devices revealed that the magneto-optic effect in our case was more sensitive to the interaction length of the flakes rather than their thickness. Consequently, we optimized our devices to balance efficient magneto-optic tuning with minimal optical losses.


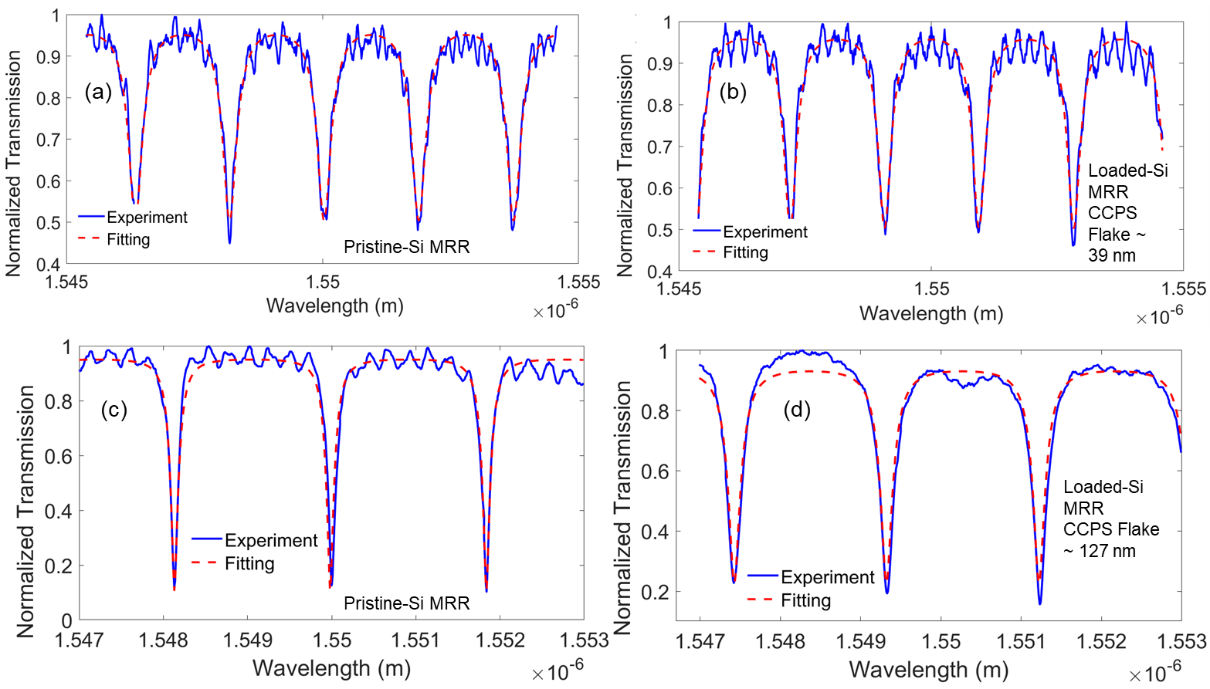


**Figure S9. Passive normalized linear** **transmission spectra of the mirroring resonator around 1550 nm for the TE mode: (a)** Reference spectrum of the bare ring. **(b)** Spectrum after loading the ring in (a) with a ~39 nm thick CCPS flake and an interaction length of 31 µm, the calculated round-trip loss (RTL) was approximately 4 dB for both the hybrid and bare silicon rings, further confirming that the presence of the 39 nm CCPS flake introduces minimal optical loss **(c)** Reference spectrum of another bare ring. **(d)** Spectrum after loading the ring in (c) with a ~127 nm thick CCPS flake and an interaction length of 58 µm.

**
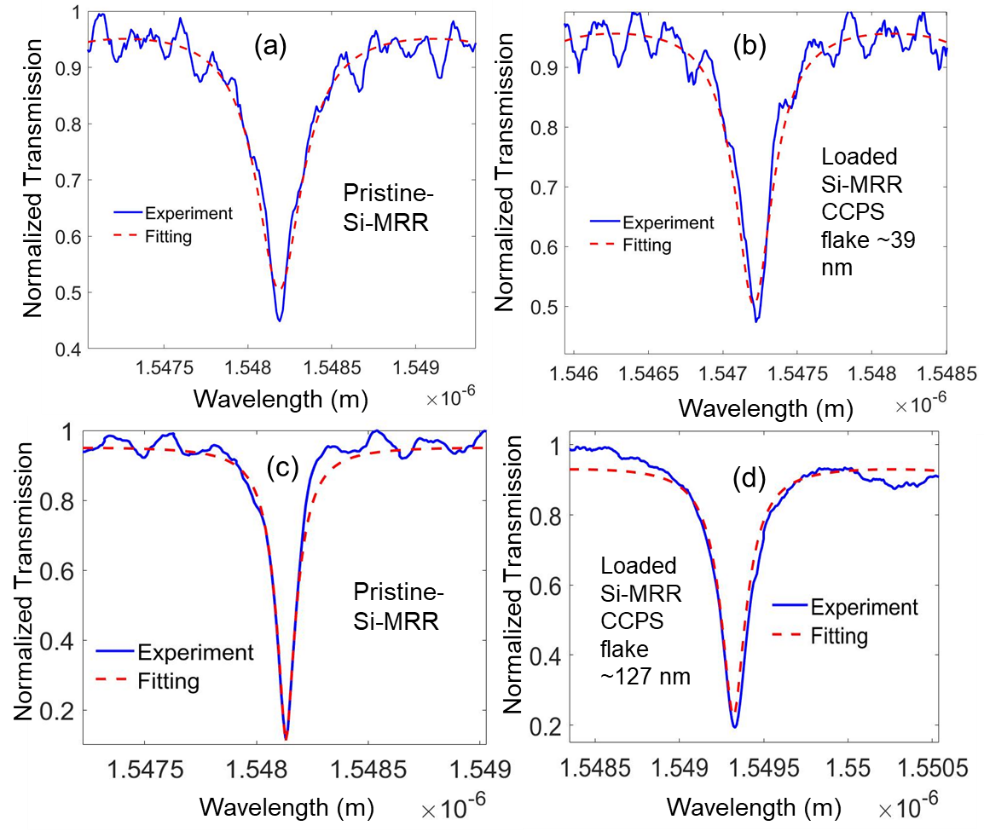
**

Close-up images of transmission spectra of the mirroring resonator around 1550 nm for the TE mode in the same order as panels in Fig. S9.

**Supplement note 2**

**Electromagnet and experimental setup**

The experimental setup for magneto-optic testing utilized a low-power DC-operated cylindrical electromagnet with a moderate lifting capacity. The electromagnet is capable of exerting a maximum initial force of 50N, stabilizing at 37N. This cylindrical electromagnet features a central core and a wiring harness. When electrical current flows through the coil of wire wrapped around the metallic core, it generates a magnetic field.

The chip was placed on the flat face of the electromagnet, ensuring that the surface of the chip was parallel to the face of the electromagnet for maximum exposure to the magnetic field. The magnetic field lines exit the north pole of the electromagnet and enter the south pole, with the strongest field being perpendicular to the surface of the disk where the sample is placed. The field lines emerge from the flat face, spread out in a loop, and return through the sides and back of the electromagnet to complete the loop.

The linearity of the electromagnet was measured using a Gauss meter, which directly recorded the magnetic field values, as shown in Fig. S10(a). In our configuration, where the photonic chip was placed directly on the flat face of the electromagnet, the primary component of the magnetic field was perpendicular to the surface of the electromagnet and the plane of the chip. Given that the chip size was smaller than the diameter of the cylindrical electromagnet, any non-uniformities in the magnetic field were negligible.

This setup is illustrated in Fig. S10(b) and (c). Figure S10(b) shows the experimental arrangement with the photonic chip side-coupled and positioned on the electromagnet stage. A close-up image of the photonic chip on the electromagnet stage is presented in Fig. S10(c), with the inset showing the magnetic field profile recorded using a magnetic field viewing film based on magnetic nanoparticles.

**
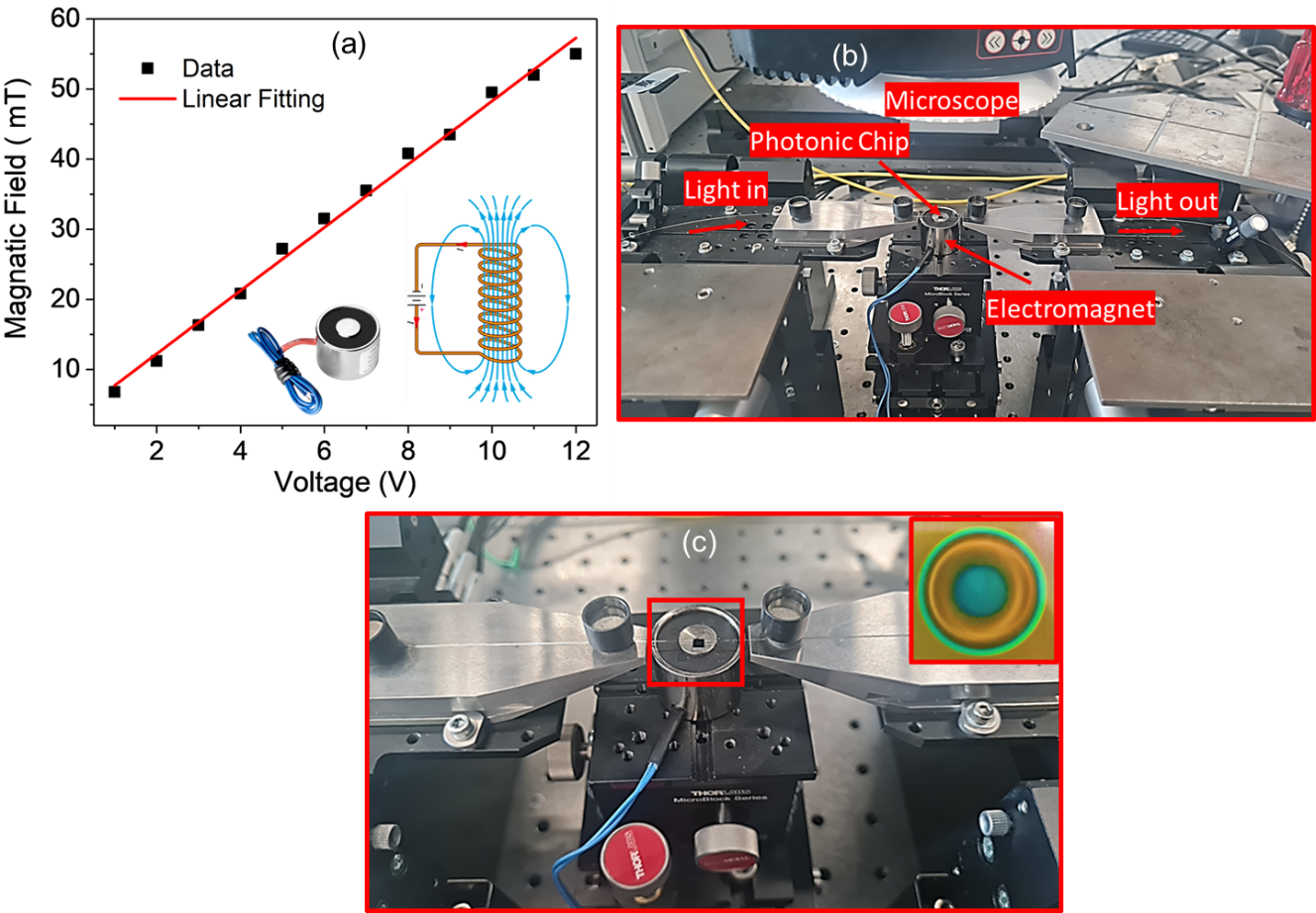
**

**Figure S10.** **Experimental setup for magneto-optic testing:** **(a)** Electromagnetic linearity as a function of DC applied voltage, with magnetic field values recorded using a Gauss meter. **(b)** Experimental setup featuring a photonic chip with side coupling, positioned on the electromagnet stage. **(c)** Close-up image of the photonic chip on the electromagnet stage, with the inset showing the magnetic field profile recorded using a magnetic field viewing film based on magnetic nanoparticles.

**
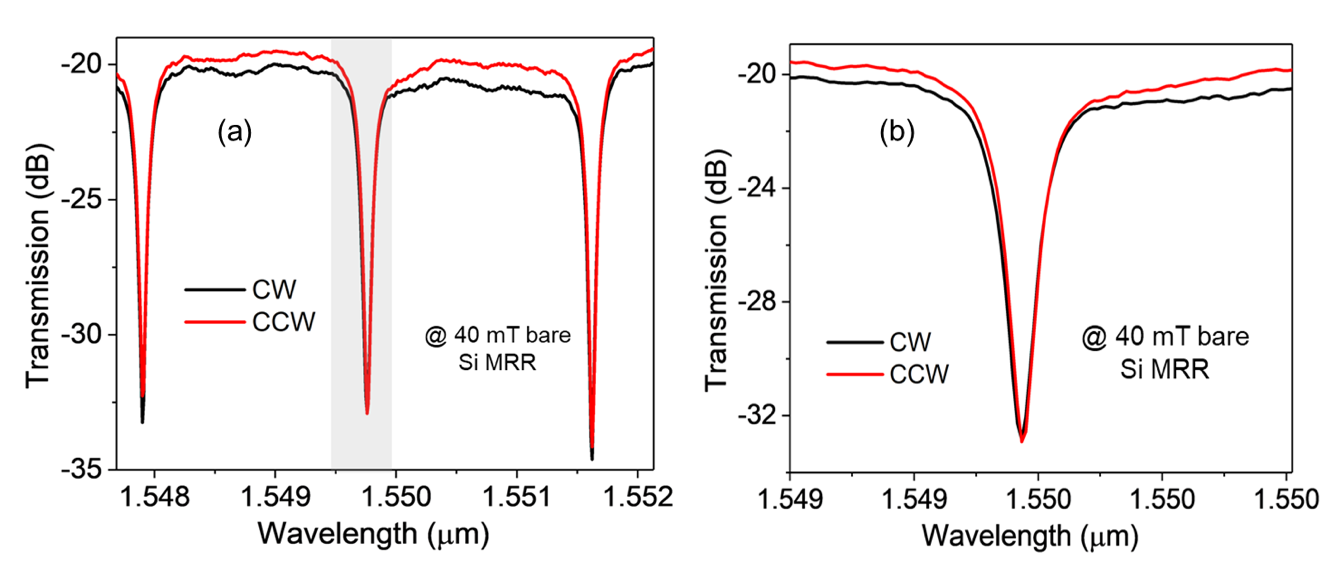
**

**Figure S11.** **Transmission spectra of the CW and CCW light propagation in a bare Si MRR under a 40 mT applied magnetic field. (a)** Wide view spectra showing three resonance peaks. **(b)** Close-up view of the peak around 1550 nm, indicating no resonance wavelength splitting (RWS) in silicon, attributed to its diamagnetic nature.

**Supplement note 3**

**Magnetic Properties and Magnetoelectric Coupling in CuCrP₂S₆ (CCPS)**

CCPS is a type-II multiferroic material that exhibits significant polarization-magnetization coupling, positioning it as a unique material for studying 2D magnetism-ferroelectricity interactions^2–4,15^. Experimental evidence has confirmed the presence of interlayer antiferromagnetism (AFM) and intralayer stripe antiferroelectricity (AFE) in its bulk form. The magnetic properties of CCPS originate from the Cr atoms, which have an incomplete 3d shell, resulting in three unpaired d electrons per atom and a corresponding magnetic moment of 3μ_B_ per formula unit. Below the Néel temperature (T_N_) of around 32 K, the Cr³⁺ magnetic moments align ferromagnetically within monolayers and are antiferromagnetically coupled between layers perpendicular to the van der Waals plane (See Fig.S12a). The ferroelectricity in CCPS is due to the Cu⁺ ions, which are randomly positioned within the ferromagnetic CrS₆-P₂S₆ cages. At 145 K, CCPS transitions to an antiferroelectric state, with Cu ions forming a striped AFE arrangement driven by a double-well pseudopotential.

Recent studies have provided further experimental and theoretical evidence of magnetoelectric coupling in CCPS, highlighting the role of spin-orbit coupling in linking electric dipoles and spins^2,3,15,16^. This coupling indicates the existence of magnetic field-induced electric polarization and electric field-controlled magnetic orderings, even in a monolayer. Although the magnetic easy axis and spin-flop transitions in CCPS are well documented^2,4^, these phenomena require further investigation for a more detailed understanding. Above the Néel temperature, CCPS is paramagnetic, with disordered spins of Cr ions. Upon cooling below T_N_, it transitions to an AFM phase with alternating interlayer spin orientations.


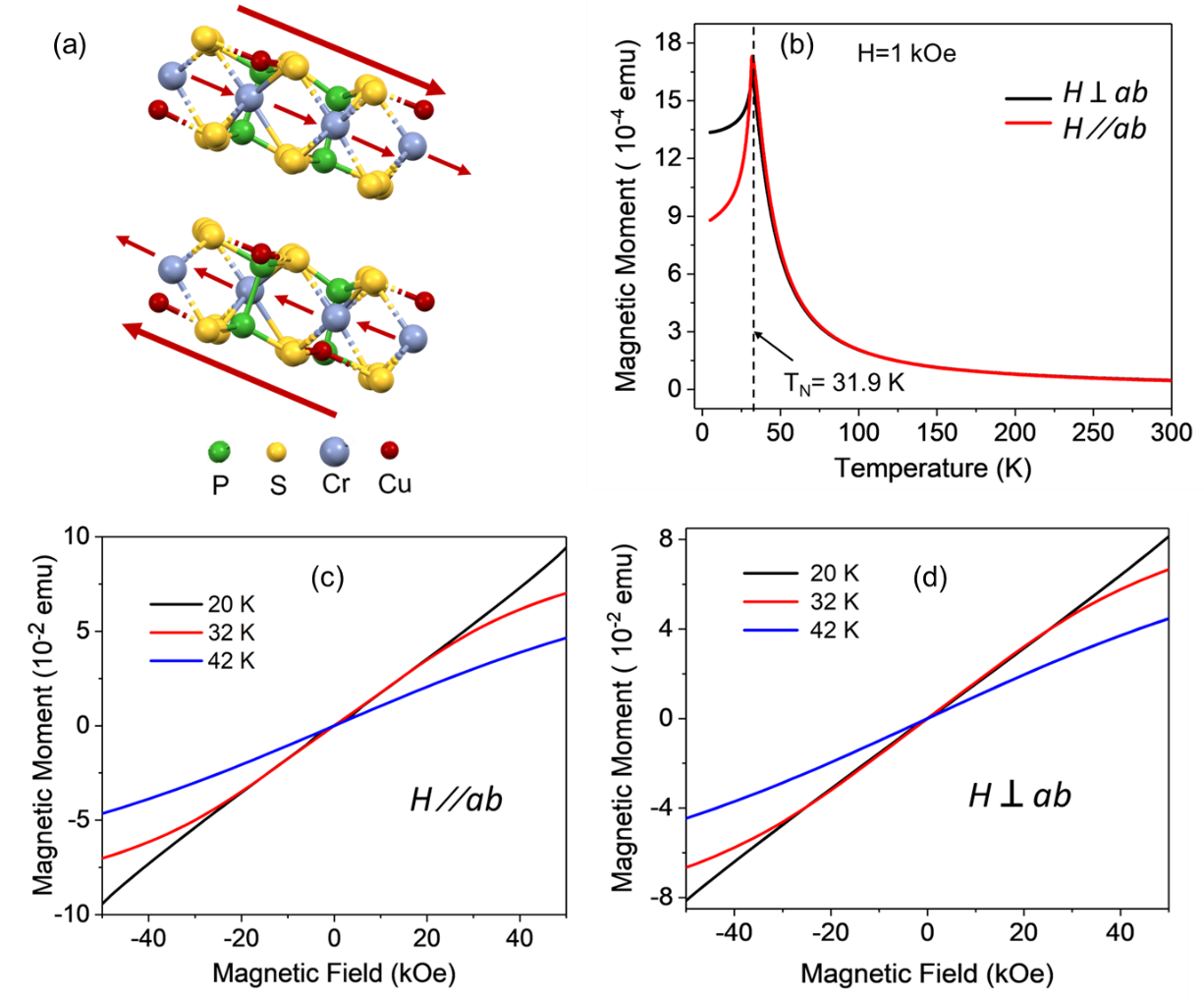


**Figure S12.** **Magnetic properties of CCPS: (a)** Schematic diagram illustrating the alignment of Cr³⁺ magnetic moments below the Néel temperature, showing ferromagnetic alignment within monolayers and antiferromagnetic coupling between layers perpendicular to the van der Waals plane. **(b)** Temperature-dependent magnetization (M-T) curves of bulk CCPS. **(c-d)** Field-dependent magnetization (M-H) curves at various temperatures with the magnetic field applied (c) parallel to the ab crystal plane and (d) perpendicular to the ab crystal plane.

**Magnetic Measurements**

The magnetic measurements were performed using a Physical Property Measurement System (PPMS) - Quantum Design DynaCool 9T. The CCPS crystal was fixed on the sample holder and oriented parallel to different crystallographic axes at various temperatures and magnetic field settings.

The temperature-dependent magnetization (*M*-*T*) of CCPS is shown in Fig. S12(b) for a magnetic field of 1 kOe applied parallel and perpendicular to the *ab* planes. As the temperature decreases from 300 K, the magnetization increases monotonically, peaking around 31.9 K, indicating a transition into an antiferromagnetically ordered state with a Néel temperature (T_N_) of 31.9 ± 1 K. A distinct cusp at T_N_ = 31.9 K is observed in both parallel and perpendicular directions, with a sharp drop in magnetization at low temperatures along the parallel direction, suggesting that the spin order favors the *ab* plane as the easy axis of magnetization. These results align well with previous studies, which reported T_N_ values of 31.5 K and 30.6 K^4,15^, underscoring the dominant ferromagnetic intralayer coupling competing with the weaker antiferromagnetic interlayer coupling in this compound.

The field-dependent magnetization (*M*-*H*) at 20 K, 32 K, and 42 K with magnetic fields applied parallel and perpendicular to the *ab* plane is shown in Fig. S12(c) and S12(d), respectively. The M-H curves in both orientations exhibit nearly linear behavior, with minimal temperature dependence. This linearity and the weak temperature dependence suggest a robust ferromagnetic intralayer coupling and a weaker antiferromagnetic interlayer interaction. The ability of the spins in CuCrP₂S₆ to be easily polarized by an applied magnetic field supports the scenario of strong ferromagnetic intralayer coupling combined with weak antiferromagnetic interlayer coupling, consistent with previous findings showing positive Curie-Weiss values^4,15^.

We conducted magnetic force microscopy (MFM) on CCPS nanoflakes of varying thickness using the NX10 Park system, which can generate an in-plane magnetic field with a maximum strength of approximately 335 Gauss (see Fig. S13). Thin flakes, specifically 20 nm and 13 nm in thickness, were selected and measured under various applied magnetic fields. The MFM results indicated that the MFM phase showed insignificant changes with the applied magnetic field. This lack of response in the MFM phase at room temperature suggests that CCPS does not exhibit ferromagnetic behavior under these conditions, indicating that the material is more likely to be paramagnetic at room temperature. However, an applied field of 335 Gauss (33.5 mT) from the bottom chuck may be insufficient to induce detectable changes in the signal.


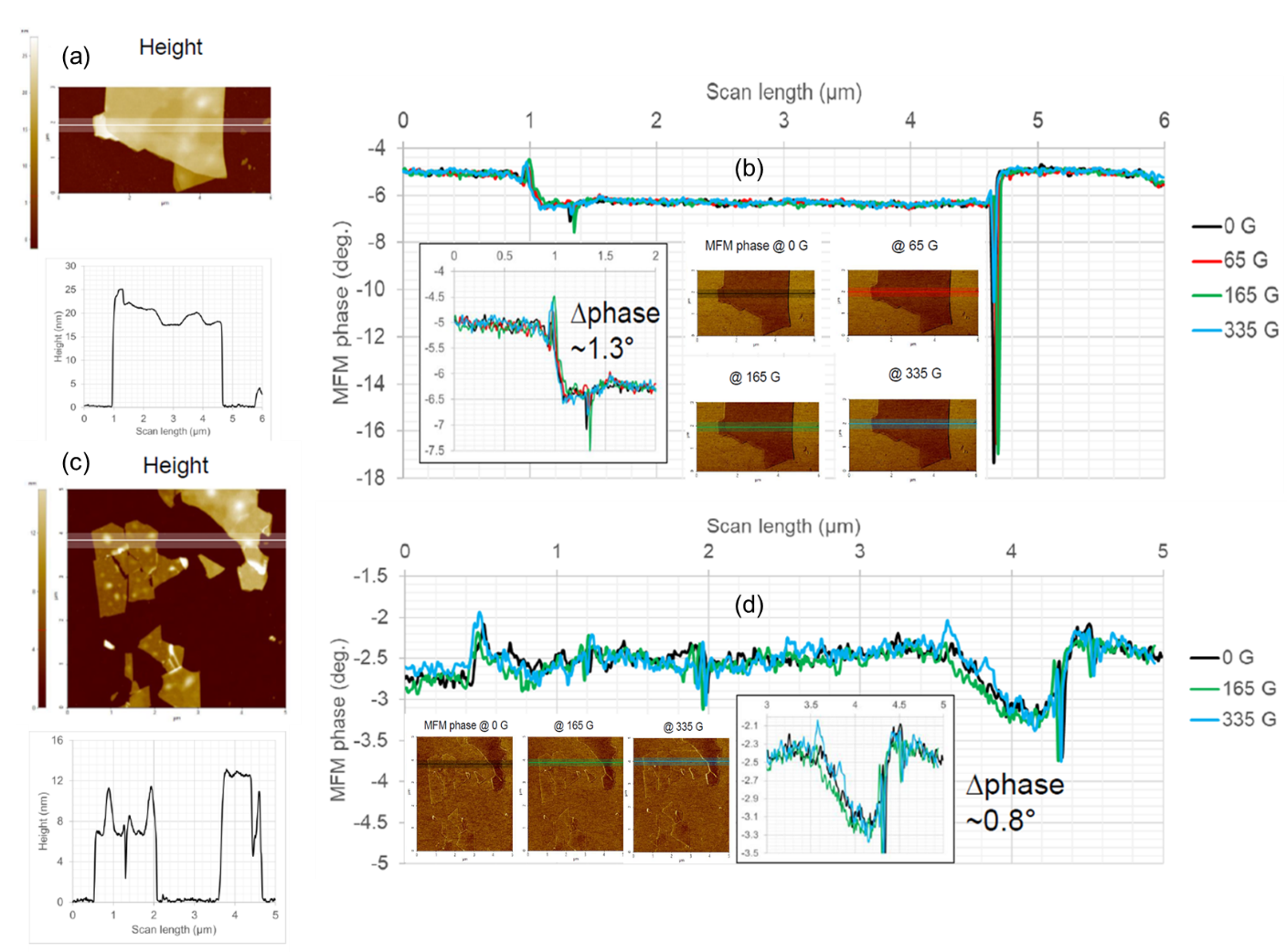


**Figure S13 Magnetic force microscopy (MFM) analysis of CCPS nanoflakes: (a)** Height sensor image displaying a nanoflake with a thickness of approximately 20 nm. **(b)** MFM phase image along lines crossing the flake, with colors indicating the applied magnetic field value. **(c)** Height sensor image displaying a nanoflake with a thickness of approximately 13 nm. **(d)** MFM phase image along lines crossing the flake, with colors indicating the applied magnetic field value.

**Supplement note 4**

**Comparative analysis, material integration and device testing**

The devices were fabricated using standard silicon-on-insulator (SOI) techniques, each chip containing 15 ring resonators. As illustrated in Fig S14(a), a pristine chip is depicted before the transfer process. Figure S14(b) provides a close-up SEM image highlighting the design details, including the 100 nm gap between the bus and the waveguide. While we did consider the use of all-pass ring resonators, our experimental results were demonstrated on an add-drop ring configuration because fabricated devices provided significantly better performance in terms of extinction ratio (ER). During fabrication, the all-pass rings exhibited lower ER and suboptimal transmission spectra, which limited their overall performance.

In contrast, the add-drop rings consistently demonstrated higher ER, better quality factors, and more reliable device operation. Given that these parameters are crucial to achieving a high isolation ratio and optimizing the overall figure of merit (FOM), we chose to focus on the add-drop configuration.

It is also worth mentioning that our fabricated devices were constructed using several photonic chips, along with various test structures. During the fabrication process, we observed some variations in the extinction ratios between different fabrication runs. To ensure accuracy, our measurements were always calibrated against bare silicon. As a reference, each ring resonator was measured both before and after the integration of CCPS. To incorporate 2D materials into the photonic chips, we developed a unique in-house transfer method, described in our previous work^17–20^.

**Deterministic transfer process**

The process began with the mechanical exfoliation of CCPS flakes using Nitto SPV224 PVC tape, followed by their transfer to a PDMS film. A gold-plated needle-based micro stamper, accurately positioned with a micro-positioner, was used to transfer the material from the PDMS. The transparency of the PDMS allowed for alignment under an optical microscope, ensuring precise flake placement on the photonic chip. Suitable flakes were identified by scanning the PDMS under a microscope, ensuring proper geometry and avoiding cross-contamination. The stamper, having a contact area larger than the flake, facilitated the precise positioning of the flake onto the target device.

This approach enabled the transfer of multiple devices. It achieves uniform CCPS layers on the rings, identifiable by consistent color contrast of the flakes, with interaction lengths ranging from 22 to 55.5 µm.


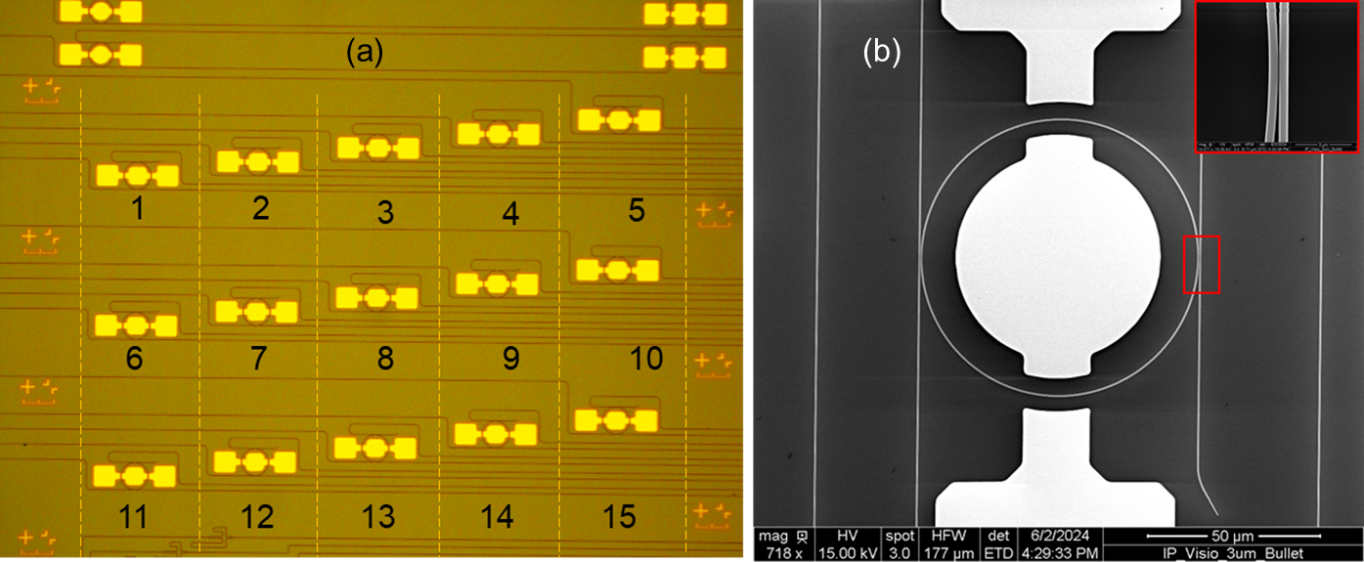


**Figure S14. Photonic chip design and fabrication: (a)** Optical microscopy image of the fabricated ring resonators prior to the transfer of CCPS, featuring 15 ring resonator devices. **(b)** SEM image providing a close-up of the fabricated resonators, with an inset highlighting the gap at the coupling region, as indicated by the red box in the resonator.

We conducted a comparative analysis to elucidate the variation in resonance wavelength splitting (RWS) and the change in effective refractive index (Δn_eff_) across devices, as influenced by the thickness and coverage length of the flakes. Figure S15(a) presents the RWS measured around 1550 nm at 40 mT and the corresponding Δn_eff_ values, calculated using Eq. 4 from the main paper, with n_g_ value of 4.4 RIU extracted from simulations. This analysis highlights the impact of flake coverage and thickness on the RWS and refractive index modulation of the devices under an applied magnetic field. Measurements from at least ten devices, with varying thickness and interaction lengths as listed in Fig. S15(b), indicate that while flake thicknesses ranging from 39 to 62 nm do not significantly affect the RWS, the interaction length of the flakes does. Figure S15(c) also shows SEM images of the various transferred devices utilized in our study.

**
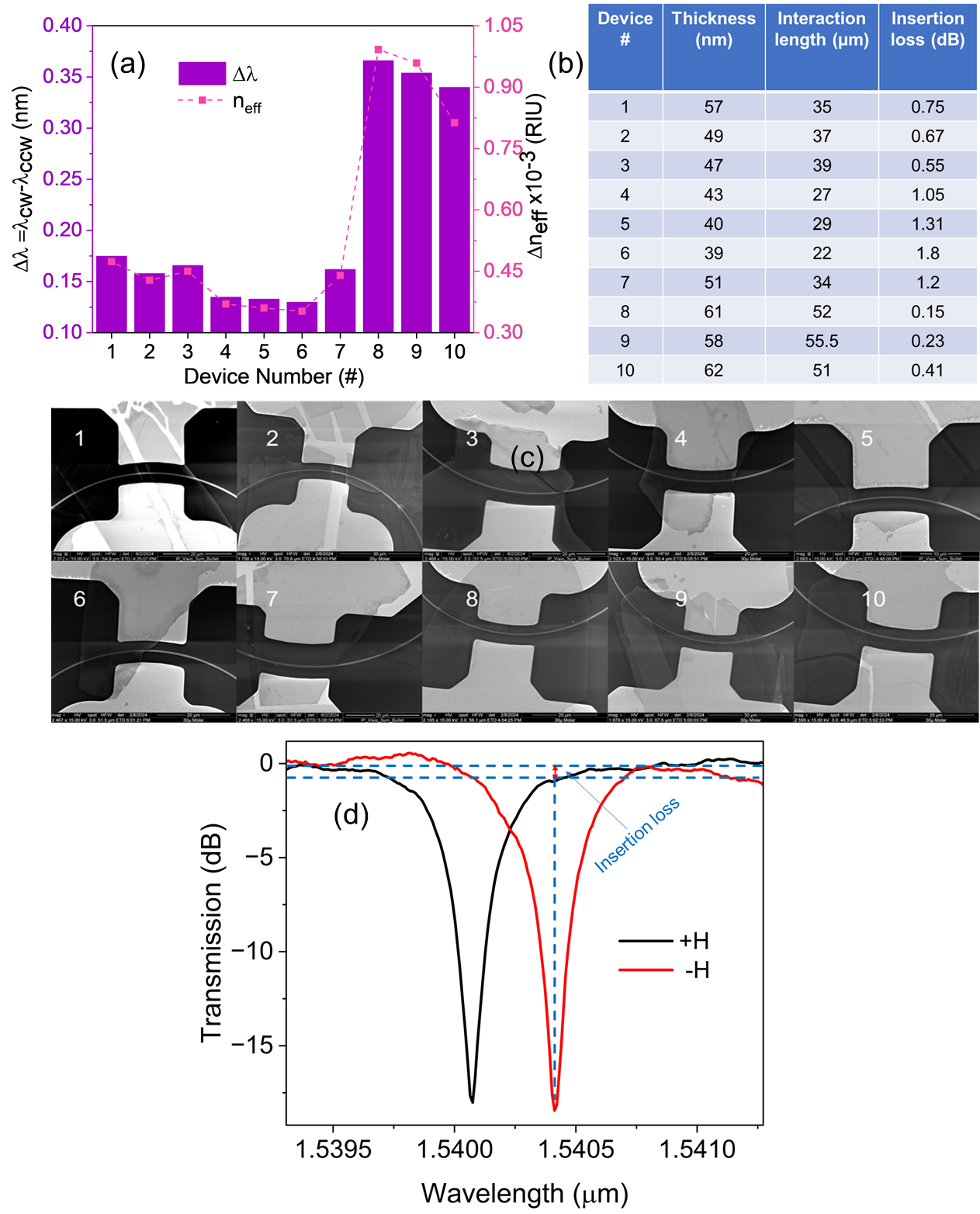
**

**Figure S15. Comparative analysis: (a)** Resonance wavelength splitting (RWS) between CW and CCW light propagation and change in effective refractive index (Δn_eff_) for 10 tested devices with thicknesses ranging from 22 nm to 62 nm and interaction lengths from 22 to 55.5 µm. **(b)** Thickness and interaction lengths of the tested devices. **(c)** Scanning electron microscopy (SEM) images of the transferred and tested devices during our experiments **(d)** The transmission spectra of one of the highest-performing integrated microring resonator (MRR) devices for forward and backward light propagation are presented, demonstrating low insertion loss. The device features a CCPS interaction length of 52 µm and an average thickness of 61 nm. Here IL= Baseline transmission (off-resonance) − Forward transmission (on-resonance at operating wavelength of the forward direction).

**Supplement note 5**

**Magneto-optic phase shifter (ΔΦ)**

**
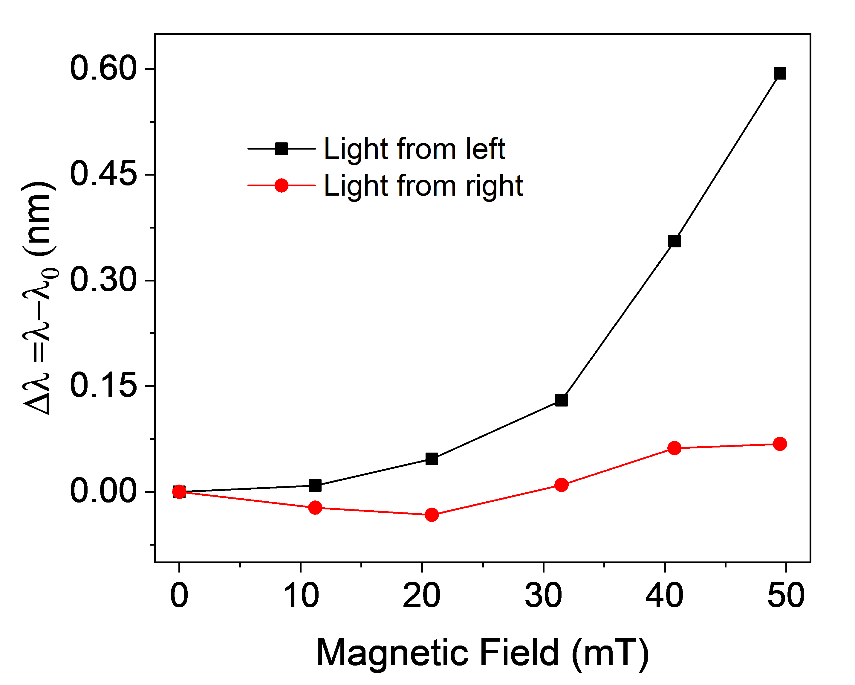
**

**Figure S16. Phase shift as a function of the magnetic field (ΔΦ).** The resonance peak position at zero magnetic field, λ₀, serves as a reference. The light was launched in either the right or left direction in two separate experiments, and the resulting phase shift (ΔΦ) was recorded as the magnetic field increased without changing its polarity. Notably, the shifts induced when the light was launched from the left differed in magnitude from those observed when launched from the right, indicating a clear deviation from the symmetric shifts expected under thermal effects.

This figure depicts the *absolute phase shift* ΔΦ, where light was launched in a **fixed direction**, either right or left, and the phase shift was recorded as the magnetic field strength increased without altering its direction. In this case, λ₀ represents the reference resonance peak at zero magnetic field.


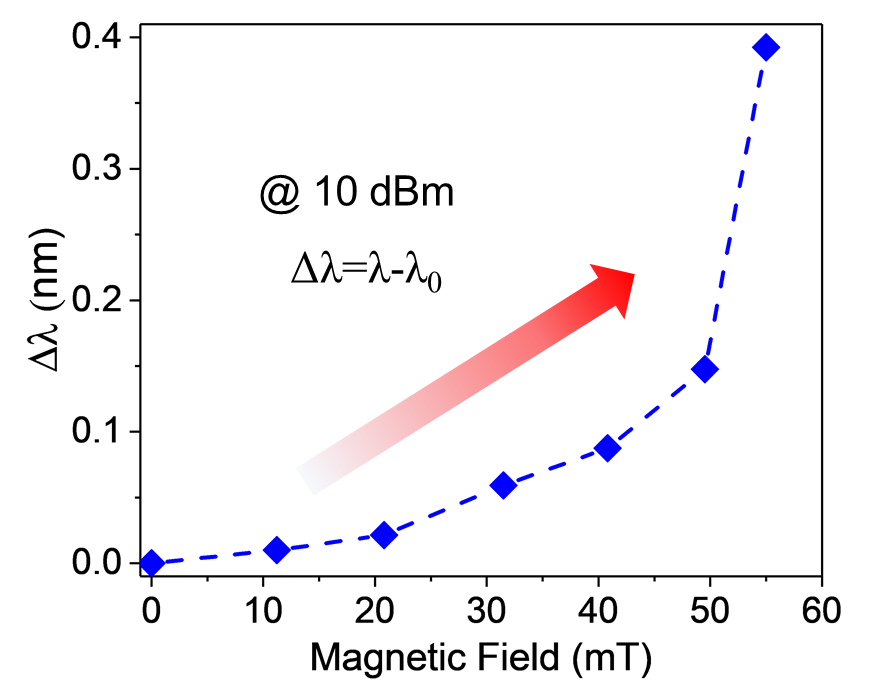


**Figure S17. Magneto-optic phase shift (ΔΦ) characteristics of the hybrid CCPS/Si micro-ring resonator (MRR). (a)** Resonance peak positions at a constant input laser power of 10 dBm, launched from the left (fixed direction), as the magnetic field varies from 0 mT to 55 mT. Here, λ₀ denotes the resonance peak position at 0 mT, and λ represents the resonance peak position at different magnetic field strengths.

It is worth noting that for light launched from the left, Figures S16 and S17 depict the same data, representing the absolute phase shift ΔΦ​. Here, λ_0_ corresponds to the reference resonance wavelength at zero magnetic field. In both figures, light was launched in a fixed direction (from the left), and the phase shift was recorded as the magnetic field strength increased without altering its direction.

The distinction between Figures S16 and S17 lies in the specific context each figure aims to highlight. Figure S16 overlays the results for light launched from both the left and right directions, illustrating the asymmetry in the induced shifts. It should be noted that in Figure S16 the data were collected separately: first, light was launched in a fixed direction (from the right), and the phase shift was recorded as the magnetic field strength was incrementally increased without altering its direction. The process was then repeated with light launched in the opposite direction (from the left), and the phase shift was similarly recorded under the same conditions.

This comparison emphasizes that the shifts for left-launched light differ in magnitude from those for right-launched light under a magnetic field. Hence, it demonstrates a clear distinction from the symmetric shifts expected due to thermal effects.

Conversely, Figure S17 isolates the data for left-launched light to focus on the nonlinear dependence of the phase shift (ΔΦ) on the applied magnetic field. This nonlinear behavior arises from the magneto-optic interaction between the optical mode and the magnetized CCPS layer, which includes contributions from the material’s nonlinear magnetization response.

For a paramagnetic material like CCPS at room temperature, the magnetization (M) as a function of the applied magnetic field (H) can be described by the Brillouin function as^21,22^:

$$M=Ng\mu_{B}JB_{J}\left( \frac{g\mu_{B}JH}{\kappa_{B}T} \right)$$

In this relation, *N* is the number of magnetic moments per unit volume, *g* is the g-factor, *μ_B_*​ is the Bohr magneton, *J* is the total angular momentum quantum number, *B_J_*_​_ is the Brillouin function, *k_B_*​ is the Boltzmann constant, *T* is the temperature. At low magnetic fields, the Brillouin function is approximately linear, and the magnetization follows$M\approx\chi H$, where $\chi$ is the magnetic susceptibility. However, as the magnetic field increases, the system enters the nonlinear regime, where the magnetization deviates from linearity and approaches saturation. For CCPS, which is well documented to exhibits magneto-electric coupling (Refs. 37, 40, 41, 43, and 44 in the manuscript), the effective *g*-factor is likely higher than the spin-only value of 2 due to enhanced spin-orbit interaction and partial unquenching of the orbital angular momentum. This increase in the *g*-factor could push the system into the nonlinear regime at 55 mT, contributing to the observed nonlinearity in the phase shift.

Importantly, the non-uniform strain in the CCPS layer, coupled with its magnetoelectric properties, is likely to further enhance the observed nonlinear response, leading to strain-induced birefringence. The nonlinear magneto-optic phase shift observed in our CCPS-integrated silicon photonic devices is consistent with similar nonlinear responses reported in other materials within the thio-selenophosphate (TSP) family, such as CuInP₂S₆ (CIPS)^23^. Previous studies have shown that CIPS exhibits significant nonlinear optical properties, including enhanced second-harmonic generation (SHG), which are highly sensitive to strain and variations in the dielectric environment. These findings suggest that the intrinsic layered structure and ferroionic characteristics of TSP materials contribute to their pronounced nonlinear optical behavior. In our case, the non-uniform coverage and inherent strain variations in the CCPS layer, combined with its magnetoelectric properties, introduce additional complexity to the light-matter interaction, resulting in a nonlinear dependence of the phase shift on the applied magnetic field. This behavior underscores the intrinsic origin of the observed nonlinearity in CCPS and suggests that such effects are characteristic of the broader TSP family, offering potential for advanced magneto-optic applications.

**Table S1 Summary of Experimental Distinctions Between Nonreciprocal and Magneto-Optic Phase Shift Measurements in the CCPS-Si Hybrid System**

| Feature | Figure 4b (Nonreciprocal Measurement) | Figures 6a, S14, S15 (Magneto-Optic Phase Shifter) |
| --- | --- | --- |
| Experimental Setup | Light alternated between CW and CCW; or switching magnetic field polarity. | Light is injected in **one direction**; magnetic field increased keeping **same polarity.** |
| Observation | Resonance splitting (Δλ) due to NRPS | Phase shift (ΔΦ) as a function of magnetic field |
| Direction Dependence | Intrinsically asymmetric (nonreciprocal) | Asymmetric ΔΦ observed between left and right light injection, excluding thermal effects |
| Physics | Nonreciprocity via differential effective indices (β+ and β-) | Magneto-optic effect causing field-dependent phase shift |
| Device Functionality | Demonstrates optical isolation potential | Demonstrates phase modulation capability |

**
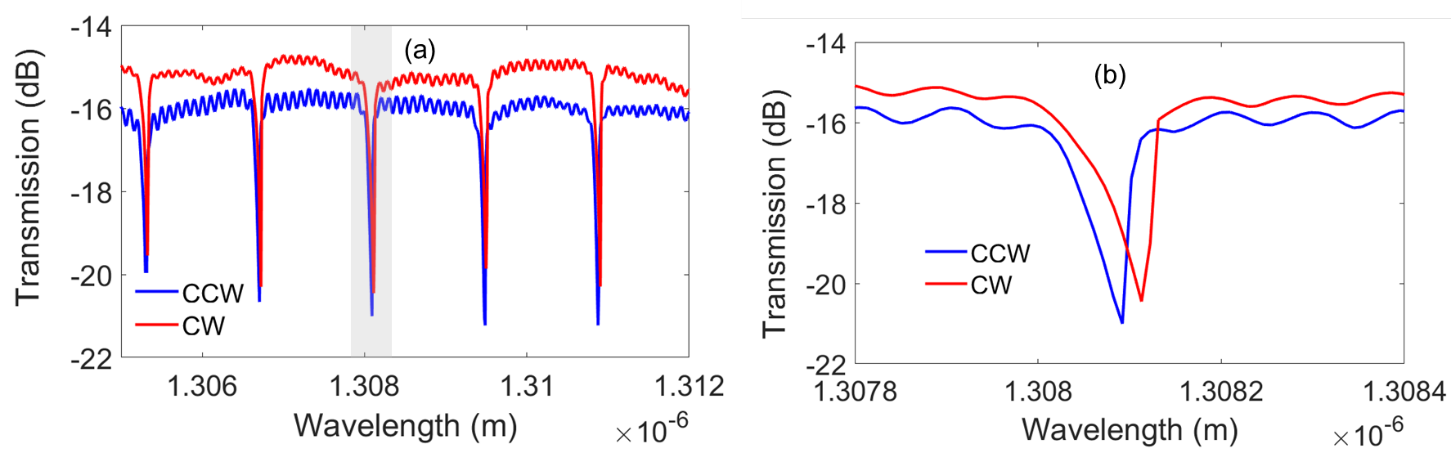
**

**Figure S18. Transmission spectra of clockwise (CW) and counterclockwise (CCW) light propagation for a 1310 nm centered laser under a 40 mT magnetic field. (a)** Wide scan view. **(b)** Close-up view highlighting the nonreciprocal response around 1310 nm.

**References**

1. Susner, M. A., Chyasnavichyus, M., McGuire, M. A., Ganesh, P. & Maksymovych, P. Metal Thio- and Selenophosphates as Multifunctional van der Waals Layered Materials. *Advanced Materials* **29**, 1602852 (2017).

2. Wang, X. *et al.* Electrical and magnetic anisotropies in van der Waals multiferroic CuCrP2S6. *Nat Commun* **14**, 840 (2023).

3. Park, C. B. *et al.* Observation of Spin‐Induced Ferroelectricity in a Layered van der Waals Antiferromagnet CuCrP _2_ S _6_. *Adv Elect Materials* **8**, 2101072 (2022).

4. Selter, S. *et al.* Crystal growth, exfoliation, and magnetic properties of quaternary quasi-two-dimensional CuCrP 2 S 6. *Phys. Rev. Materials* **7**, 033402 (2023).

5. Maiti, R. *et al.* Strain-engineered high-responsivity MoTe2 photodetector for silicon photonic integrated circuits. *Nat. Photonics* **14**, 578–584 (2020).

6. Carrascoso, F. *et al.* Improved strain engineering of 2D materials by adamantane plasma polymer encapsulation. *npj 2D Mater Appl* **7**, 1–8 (2023).

7. Aslan, B. *et al.* Probing the Optical Properties and Strain-Tuning of Ultrathin Mo1–xWxTe2. *Nano Lett.* **18**, 2485–2491 (2018).

8. Yan, W. *et al.* On-Chip Nonreciprocal Photonic Devices Based on Hybrid Integration of Magneto-Optical Garnet Thin Films on Silicon. *IEEE J. Select. Topics Quantum Electron.* **28**, 1–15 (2022).

9. Moss, D. Optical isolator and power limiter in silicon nanowires with 2D graphene oxide films. (2024).

10. Ma, J., Xi, X., Yu, Z. & Sun, X. Hybrid graphene/silicon integrated optical isolators with photonic spin–orbit interaction. *Applied Physics Letters* **108**, 151103 (2016).

11. Zheng, J. *et al.* GST-on-silicon hybrid nanophotonic integrated circuits: a non-volatile quasi-continuously reprogrammable platform. *Opt. Mater. Express* **8**, 1551 (2018).

12. Wei, G., Stanev, T. K., Czaplewski, D. A., Jung, I. W. & Stern, N. P. Silicon-nitride photonic circuits interfaced with monolayer MoS2. *Applied Physics Letters* **107**, 091112 (2015).

13. Rabiei, P., Steier, W. H., Cheng Zhang & Dalton, L. R. Polymer micro-ring filters and modulators. *J. Lightwave Technol.* **20**, 1968–1975 (2002).

14. Miller, S. A. *et al.* Low-loss silicon platform for broadband mid-infrared photonics. *Optica* **4**, 707 (2017).

15. Kleemann, W., Shvartsman, V. V., Borisov, P., Banys, J. & Vysochanskii, Yu. M. Magnetic and polar phases and dynamical clustering in multiferroic layered solid solutions CuCr 1 − x In x P 2 S 6. *Phys. Rev. B* **84**, 094411 (2011).

16. Hu, Q. *et al.* Ferrielectricity controlled widely-tunable magnetoelectric coupling in van der Waals multiferroics. *Nat Commun* **15**, 3029 (2024).

17. Dushaq, G., Paredes, B., Villegas, J. E., Tamalampudi, S. R. & Rasras, M. On-chip integration of 2D Van der Waals germanium phosphide (GeP) for active silicon photonics devices. *Opt. Express* **30**, 15986 (2022).

18. Dushaq, G., Villegas, J. E., Paredes, B., Tamalampudi, S. R. & Rasras, M. S. Anisotropic Van Der Waals 2D GeAs Integrated on Silicon Four-Waveguide Crossing. *J. Lightwave Technol.* **41**, 1784–1789 (2023).

19. Tamalampudi, S. R., Dushaq, G., Villegas, J. E., Paredes, B. & Rasras, M. S. A Multi-layered GaGeTe Electro-Optic Device Integrated in Silicon Photonics. *J. Lightwave Technol.* 1–7 (2023) doi:10.1109/JLT.2023.3237818.

20. Tamalampudi, S. R. *et al.* High-Speed Waveguide-Integrated InSe Photodetector on SiN Photonics for Near-Infrared Applications. *Advanced Photonics Research* **4**, 2300162 (2023).

21. Ding, X. *et al.* Anomalous paramagnetism in graphene on hexagonal boron nitride substrates. *Phys. Rev. B* **84**, 174417 (2011).

22. Kao, C.-W. *et al.* Role of Fe-Doping Effect in 2-D MoS2 Magnetic Semiconductor. *IEEE Transactions on Magnetics* **54**, 1–3 (2018).

23. Rahman, S., Yildirim, T., Tebyetekerwa, M., Khan, A. R. & Lu, Y. Extraordinary Nonlinear Optical Interaction from Strained Nanostructures in van der Waals CuInP2S6. *ACS Nano* **16**, 13959–13968 (2022).
